# Leukotrienes: Bridging the Inflammatory Gap in Asthma and Inflammatory Bowel Diseases (IBD)

**DOI:** 10.1002/cph4.70022

**Published:** 2025-06-26

**Authors:** Emma Elizabeth Sabu Kattuman, Lakshminarayan Reddy Teegala, Somayeh Darzi, Charles K. Thodeti, Sailaja Paruchuri

**Affiliations:** ^1^ Department of Physiology and Pharmacology The University of Toledo College of Medicine and Life Sciences Toledo Ohio USA

**Keywords:** asthma, chronic inflammation, inflammatory bowel disease, inter‐organ communication, leukotrienes, lung–gut axis

## Abstract

Leukotrienes are potent inflammatory lipid mediators produced primarily by immune cells. Inflammation, being the center stone of two major disease conditions, namely, asthma and inflammatory bowel disease (IBD), has led researchers to study the role of leukotrienes (LTs) in both these disease settings extensively. Several studies indicate a crucial role for LTs in the development and progression of IBD, whereas LTs, especially cysteinyl leukotrienes (cys‐LTs), have been identified as the major contributors to asthma initiation and progression for over three decades. Additionally, the lungs and the gut share several common characteristics, including their exposure to the external environment, similar microbiome composition, and inflammatory responses. These similarities suggest a bidirectional relationship, supported by the increased risk of IBD in asthma patients and vice versa. However, the specific role of LTs in this lung–gut connection remains unclear. This review will examine how several common factors, such as physiology, microbiome, environment, and inflammatory mediators, especially LTs, modulate this crosstalk. The review also highlights in detail how altered leukotriene biosynthesis and signaling contribute to the pathogenesis of both asthma and IBD. Furthermore, we will consider the therapeutic implications of targeting leukotriene pathways for patients with concurrent asthma and IBD in the hope of developing more efficient treatment outcomes for these interconnected conditions. Finally, this review will very briefly explore the involvement of neuronal connections in mediating the lung–gut crosstalk.

## Graphical abstract

Dissecting the pathophysiological role of Leukotrienes in the lung–gut axis: A potential mediator of the asthma–IBD crosstalk.

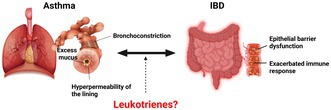

## 1 Introduction

A total of 8.7% of the American population has been diagnosed with chronic asthma in 2024 (Swed et al. 2024). Among these, almost 5%–10% of the patients are treatment‐resistant and suffer from chronic asthmatic symptoms that disrupt their daily life (Narasimhan 2021). This number indicates a major challenge in discovering effective treatment options for patients with treatment‐resistant chronic asthma. The most common symptoms observed during asthma exacerbation are wheezing, shortness of breath, coughing, chest congestion, and fatigue, which are primarily caused by chronic airway obstruction. Chronic inflammation of the lower respiratory tract, the hallmark of asthma, can be triggered by various environmental and emotional factors, including pollen, dust mites, air pollutants, certain food additives, and strong emotions. The persistent inflammatory response leads to airway wall thickening, mucus secretion and deposition, and airway remodeling in humans (Gans and Gavrilova 2020). Similar to asthma, persistent inflammation is the key player in the initiation of another inflammatory condition called inflammatory bowel disease (IBD), specifically, ulcerative colitis (UC). UC is characterized by the chronic inflammation of the large intestine starting at the rectum and extending throughout or partially across the colon. UC can be triggered through a complex interaction of genetic, autoimmune, and environmental factors that trigger an exacerbated immune response against the colonic mucosal surface (Ordas et al. 2012). Erosion of the mucus wall, diarrhea with bloody stools, increased susceptibility to developing colon cancer, and rectal spasms are the most common disease manifestations in patients with UC (Di Sabatino et al. 2012). Epidemiological insights reveal that close to 1 million people in the United States are living with UC. Interestingly, while girls show higher susceptibility to UC in children, adult males are 1.5 times more likely to develop UC than females (Rustgi et al. 2020). Chronic UC is a lifelong condition that requires ongoing management. Importantly, incomplete remission to current treatment strategies mandates the need for better therapeutic targets.

Many studies in the past have shown similarities between the pathophysiology and biochemistry of obstructive pulmonary diseases like asthma and UC, suggesting a possible bidirectional relationship between the two conditions, known as the “lung–gut axis”. This is further supported by the increased susceptibility of patients with obstructive pulmonary diseases such as asthma to develop UC or Crohn's disease later in their life and vice versa, providing compelling evidence for a crosstalk between these two conditions (Brassard et al. 2015; Ekbom et al. 2008; Hemminki et al. 2010; Keely et al. 2012; Kuenzig et al. 2017; Labarca et al. 2019; Virta et al. 2013; Zou et al. 2023; Douglas et al. 1989; Eade et al. 1980; Jacobsen et al. 2024; Louis et al. 1995; Marvisi et al. 2000; Miehsler et al. 2004; Pasquis et al. 1981; Songur et al. 2003; Tzanakis et al. 1998; Yilmaz et al. 2010; Papanikolaou et al. 2014). This review aims to consolidate evidence from various studies supporting the existence of the lung–gut axis while detailing some of the known mediators of this axis. It will broadly explore how alterations in environmental, microbial, and immunological influences contribute to the pathogenesis of both asthma and IBD. Along with the inflammatory mediators that have already been shown to be common between the two conditions, this review will also examine the role of leukotrienes (LTs) in linking asthma and IBD, especially UC, for the first time. LTs are bioactive lipid mediators derived from membrane phospholipids via the arachidonic acid cascade. Since LTs are highly implicated in both asthma (Singh et al. 2013) and UC (Schumert et al. 1988; Sklyarov et al. 2011) individually, we suspect that they could be one of the common links between the two conditions. Additionally, we will explore the potential of targeting leukotriene pathways as a promising avenue for developing more effective treatments for patients suffering from both asthma and IBD. Finally, we will discuss some recent studies that have implicated the possible neuro‐immune connections linking the gut and lung crosstalk.

### 1.1 The “Lung–Gut Bidirectional Axis”

**FIGURE 1:**
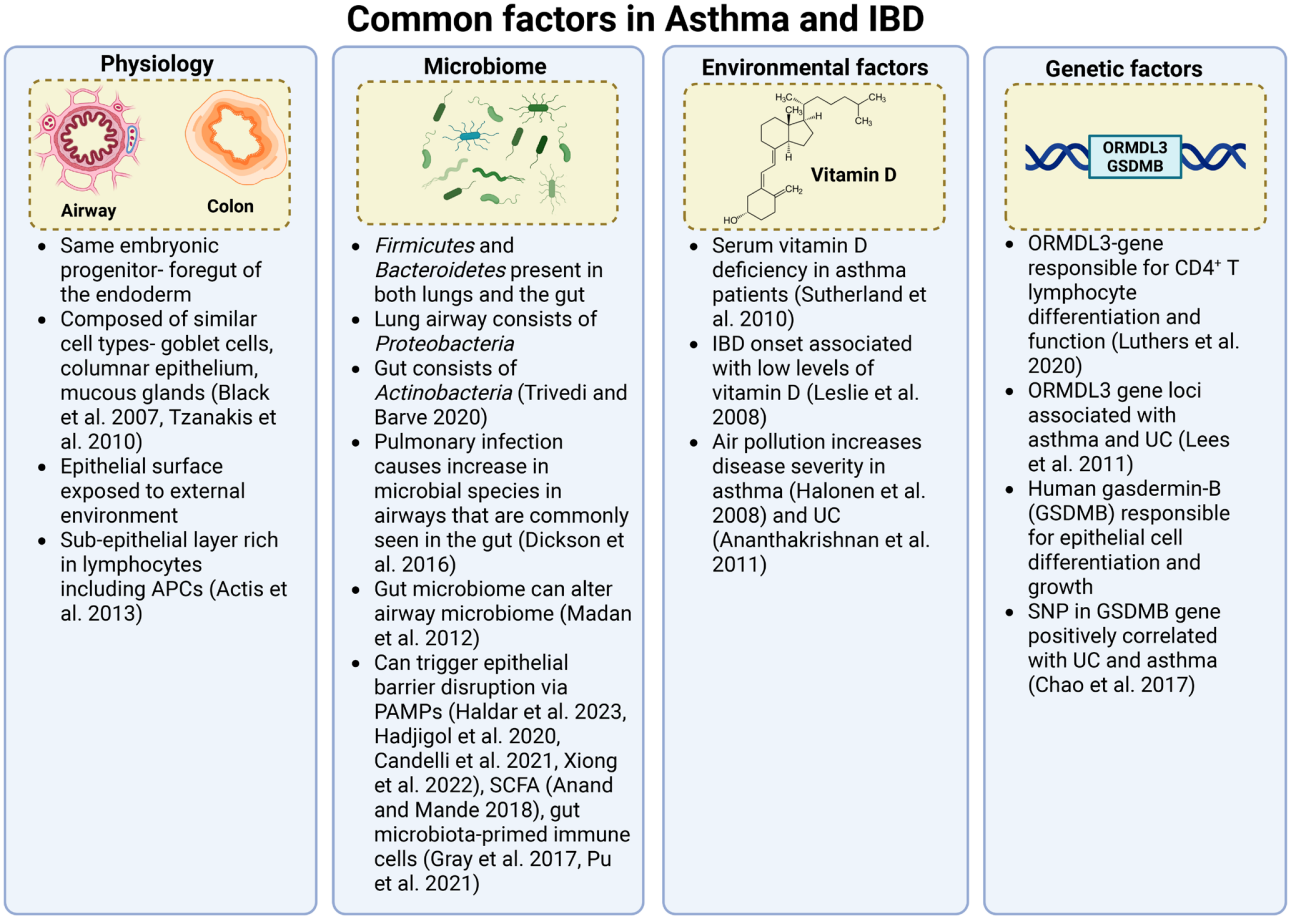
Schematic representation of the common factors in asthma and IBD. Figure created with Biorender.com.

The concept of the “lung–gut axis” has gained significant attention over the past decade, with multiple studies exploring the common links and biochemical pathways involved. Findings by Warwick Turner in 1968 laid the groundwork for subsequent studies on lung–gut bidirectional crosstalk (Turner‐Warwick 1968). A case report from 1976 discovered severe bronchopulmonary disease in six IBD patients, suggesting pulmonary complications as a manifestation of IBD (Kraft et al. 1976). Further, Ceyhan et al. in 2003 reported higher incidence of allergic reactions, pulmonary discomfort, and abnormal lung function tests in IBD patients (Ceyhan et al. 2003). Asthma, a pulmonary complication that is the focus of this review, was also shown to be more prevalent in patients with both UC and Crohn's disease (Bernstein et al. 2005). A population‐based cohort study of 1547 UC patients revealed a sixfold increase in asthma‐related deaths (Persson et al. 1996). In the subsequent years, multiple studies unraveled the high prevalence of IBD patients with chronic pulmonary complications indicating an association from the gut to the lung (Douglas et al. 1989; Eade et al. 1980; Jacobsen et al. 2024; Louis et al. 1995; Marvisi et al. 2000; Miehsler et al. 2004; Pasquis et al. 1981; Songur et al. 2003; Tzanakis et al. 1998; Yilmaz et al. 2010; Papanikolaou et al. 2014; Persson et al. 1996). However, in the past two decades, there has been emerging evidence of increased IBD susceptibility as a complication of pulmonary obstruction (Brassard et al. 2015; Ekbom et al. 2008; Hemminki et al. 2010; Keely et al. 2012; Kuenzig et al. 2017; Labarca et al. 2019; Virta et al. 2013; Zou et al. 2023). Therefore, it is now accepted that the association between the lung and the gut is rather bidirectional. Table 1 summarizes various studies indicating a link between pulmonary complications (including asthma) and IBD in humans. Common physiological and biochemical factors may link the two conditions. This review aims to explore factors common to asthma and IBD that may serve as connecting links, with a focus on pro‐inflammatory lipid mediators like LTs. Some of the common factors are as follows, detailed in Figure 1.

**TABLE 1:** Some literature evidence for lung–gut crosstalk in asthma and IBD.

References	Summary	PMID
Kraft et al. (1976)	Bronchopulmonary disease in six pre‐diagnosed IBD patients	1267553
Ceyhan et al. (2003) Louis et al. (1995)	Bronchial hyperreactivity, abnormal pulmonary tests, and respiratory complications commonly seen in IBD patients	12584393 8546267
Bernstein et al. (2005)	Both Crohn's and UC patients expressed increased susceptibility to develop asthma	16143122
Persson et al. (1996)	Six‐fold increase in no. of deaths in UC patients also diagnosed with asthma	8613037
Songur et al. (2003)	80% of 36 patients cohort experiencing pulmonary complications had active bowel disease	2616823
Eade et al. (1980)	Abnormal pulmonary function test in IBD patients, Low carbon monoxide transfer factor in patients than controls	6104919
Marvisi et al. (2000)	Abnormal pulmonary function tests are more observed in active IBD patients compared to inactive patients, and the reduction in carbon monoxide diffusing factor	11153600
Jacobsen et al. (2024)	Increased risk of developing asthma and other Obstructive Lung Disease (OLD) for IBD patients in Danish patient cohort	38183388
Hemminki et al. (2010)	Crohn's disease was significantly severe in asthma patients in a Swedish population study	20036578
Kuenzig et al. (2017)	Asthma was associated with early and late‐onset ulcerative colitis in Canadian patient cohort	28344063
Brassard et al. (2015)	Incidence of IBD increased in asthma patients compared to general population of Quebec, Canada	25406447
Zou et al. (2023)	Adult‐onset asthma is associated with increased risk of UC diagnosis and IL‐18 may play a causal role	38162651
Clarke et al. (2018)	IBD and asthma treatment focused on anti‐TLA1 antibody	29436901

#### 1.1.1 Physiology of the Lungs and the Gut

The lungs and the gut share several physiological features despite their anatomical distance. They both are embryonically derived from the same progenitor—the foregut portion of the endoderm. Both the lungs and the gut are composed of similar cell types, including goblet cells, mucous glands, and with columnar epithelium (Black et al. 2007; Tzanakis et al. 2010). The outermost mucosal epithelia in both the lungs and the gut are constantly exposed to the external environment. As a defense mechanism, the submucosal layer of the lungs and the gut consists of a high number of lymphocytes, including antigen‐presenting cells, which contributes to the cytokine release during infectious attacks (Actis and Rosina 2013). Furthermore, the mucus secretion in both the organs is influenced by their respective microbiome, and disruption of the mucosal layer has been implicated in both asthma and UC (Hansson 2019; Welsh et al. 2017). These shared physiological characteristics contribute to the potential for a bidirectional relationship between lung and gut health in conditions like asthma and UC.

#### 1.1.2 Microbiome

Although the gut and the respiratory tract microbiota share similarities at the phylum level, the dominant members within these phyla are different. While *Firmicutes* and *Bacteroidetes* are predominant in both the respiratory airway and the gut, *Actinobacteria* colonize mainly in the gut, whereas *Proteobacteria* colonize in the airway (Trivedi and Barve 2020). Interestingly, a study by Dickson et al. in 2016 described that following the events of septic shock and acute respiratory distress syndrome (ARDS), the microbial population in the lungs gets enriched with *Bacteroides* spp. that are commonly found in the gut microbiome (Dickson et al. 2016). Similarly, Madan et al. suggested that changes in the gut microbiome could have an impact on or alter the respiratory microbiome (Madan et al. 2012). These results emphasize microbial composition as one of the key factors connecting the lung and the gut. Notably, exposure to antibiotics that disrupt the gut microbiota in the first six months could lead to the development of asthma and allergies by the age of six (Risnes et al. 2011). Along similar lines, Le and Burks in 2014 found that antibiotic exposure in the first 2 years of life could cause asthma development by the age of seven and a half (Le and Burks 2014). Exposure to antibiotics can deplete the microbial population, which has been shown to be essential for T helper cell 1 (Th1) maturation and T helper cell 2 (Th2) response, two immune components that play a crucial role in allergy (Modi et al. 2014).

Gut microbiota can communicate and alter the lung microbiome through various mechanisms. Some of these mechanisms include

1. The secretion of pathogen‐associated molecular patterns (PAMPs), including peptidoglycans and lipopolysaccharides (LPS), into systemic circulation (Haldar et al. 2023). This communication occurs via Toll‐like receptors (TLRs), which can activate various immune cells in response to PAMPs. Exacerbated activation of TLRs by PAMPs can trigger immune hyperresponsiveness leading to a cytokine storm that disrupts the epithelial barrier in IBD and pulmonary diseases. Although low doses of LPS exposure in early childhood are protective against allergy and asthma (due to suppression of Th2 immune response) (Qian et al. 2018), high levels of LPS exposure worsen the condition by increasing the number of infiltrating neutrophils and macrophages and their release of inflammatory cytokines (Hadjigol et al. 2020). Multiple studies, including the recent study by Xiong et al., point out the pathogenic effect of LPS on the development and progression of UC (Candelli et al. 2021; Xiong et al. 2022). Interestingly, the bacterial LPS/TLR4 axis has been reported to stimulate IL‐13 production in mast cells (MCs) via the leukotriene B_4_ receptor, BLT2, promoting allergic response (Lee et al. 2017).
2. The secretion of secondary metabolites such as short‐chain fatty acids (SCFAs). SCFAs, which are products of dietary fiber digestion by the gut microbiota, mainly consist of butyrate, propionate, and acetate. These SCFAs produced in the gut, absorbed by colonic epithelial cells, and secreted into the systemic circulation can in turn alter the lung microbiota by modulating the immune environment and maintaining mucosal barrier integrity (Anand and Mande 2018; Furusawa et al. 2013). SCFAs, especially butyrate, have been shown to inhibit histone deacetylase (HDAC) activity, thereby promoting the expression of the Foxp3 gene, a key regulator of regulatory T cell differentiation and activation (Furusawa et al. 2013). Our lab further validated the beneficial effects of butyrate on MC activation, a key process in the initiation and progression of asthma. Our data demonstrated that butyrate inhibits stem cell factor (SCF)‐mediated MC inflammatory responses and proliferation by inhibiting HDAC1/3 activity (Gudneppanavar et al. 2023). Along with butyrate, another SCFA, propionate, has also been shown to exert beneficial effects in allergic inflammation. Propionate alters bone‐marrow hematopoiesis, leading to an increased number of macrophage and dendritic cell (DC) precursors in the lungs. Notably, these DCs are highly phagocytic but are impaired in their ability to promote Th2‐mediated inflammation (Trompette et al. 2014). SCFAs have also been shown to maintain mucosal barrier integrity, promote the survival of commensal microbes, and modulate immune responses in the lung (Anand and Mande 2018). Increased levels of SCFAs in the gut have been correlated with alleviating pulmonary disorders as well as IBD (Anand and Mande 2018). Importantly, our food intake can impact gut microbiota composition, which in turn determines the differential SCFA secretion in the colon, influencing IBD and/or asthma. For example, plant proteins increase the levels of *Lactobacillus* and *Bifidobacteria*, both of which cause an increase in the levels of SCFAs in the gut. In contrast, animal proteins have been shown to increase the levels of *Bacteroides* and *Alistipes*, which reduce SCFAs in the gut (Anand and Mande 2018). Notably, unsaturated fats such as fish oil rich in omega‐3 fatty acids increased the levels of *Roseburia*, *Ruminococcus*, and *Eubacterium* in the gut, leading to increased levels of fecal butyrate, offering protection from asthma, pneumonia, and COPD (Anand and Mande 2018). However, saturated fats such as lard fat have an opposite effect by reducing the levels of *Bifidobacteria* and *Eubacterium* and decreasing SCFA levels in the gut (Anand and Mande 2018).
3. The direct transport of whole bacteria or bacterial components to the lungs. Bacterial translocation from the gut to the lung or the upper oral cavity can occur as a result of aspiration (Budden et al. 2017). This is further supported by the observation that lung microbiota after sepsis or ARDS gets enriched with the gut microbiome (Dickson et al. 2016).
4. The translocation of gut microbiota‐primed immune cells as well as immune modulators to the lungs. Immune cells such as the innate lymphoid cells type 2 and type 3 (ILC2 and ILC3) can migrate from the gut to the lungs based on cues from the gut microbiota via systemic circulation (Gray et al. 2017; Pu et al. 2021). While ILC2s play a role in maintaining mucosal barrier integrity, ILC3s are important for allergic anti‐pathogenic responses. Additionally, gut microbiota, particularly *Candida* species, can also influence the secretion of immune mediators such as prostaglandins, especially PGE_2_, from immune cells (Underhill and Iliev 2014). The secreted PGE_2_ can then travel to the lungs through blood and polarize macrophages, leading to allergic inflammation (Kim et al. 2014). These studies support the role of the microbiome in both the lung and the gut during asthma and IBD.

#### 1.1.3 Environmental and Genetic Factors

Vitamin D, capable of cytokine inhibition and enhancing innate immunity, was observed to be deficient in both asthma (Sutherland et al. 2010) and IBD patients (Leslie et al. 2008). Notably, air pollution increases disease severity in both asthma (Halonen et al. 2008) and UC (Ananthakrishnan et al. 2011). Besides environmental factors, the ORMDL3 gene loci was found to be associated with both UC and asthma through a genome‐wide association study (Lees et al. 2011). ORMDL3 influences the differentiation and function of CD4^+^ T lymphocytes and can affect the production of cytokines and the activation of various immune cells (Luthers et al. 2020), contributing to the lung and gut crosstalk. Human gasdermin‐B (GSDMB), broadly known to regulate the differentiation and growth of epithelial cells, is yet another gene that has been shown to undergo single nucleotide polymorphism (SNP) and positively correlate with UC as well as asthma (Chao et al. 2017). Although there are limited to no studies directly depicting SNPs in genes involved in leukotriene synthesis and signaling in association with IBD, numerous pieces of literature points toward the genetic alterations in the leukotriene pathway that manifest asthma in humans. Genetic mutations, such as variable number tandem repeats (VNTR) in the Sp1‐binding motif of the *Alox5* gene (which encodes for the 5‐lipooxygenase enzyme) are associated with enhanced bronchoconstriction and leukotriene load (Thompson et al. 2016). Further, a 444A > C SNP polymorphism in the *LTC*_*4*_ *S* gene (encodes for leukotriene C_4_ synthase enzyme) can potentiate asthma risk along with reduced sensitivity toward CysLT_1_R antagonist, Zafirlukast. Additionally, the 601 A > G variant of the *Cysltr2* gene, encoding the Met201Val CYSLT_2_R receptor variant, is found among the European population and is associated with atopic asthma. The Gly300Ser CYSLT_1_R variant is yet another SNP of the *Cysltr1* gene shown to be associated with atopic asthma [leukotriene synthesis and signaling pathway explained in the later sections].

#### 1.1.4 Inflammation

**FIGURE 2:**
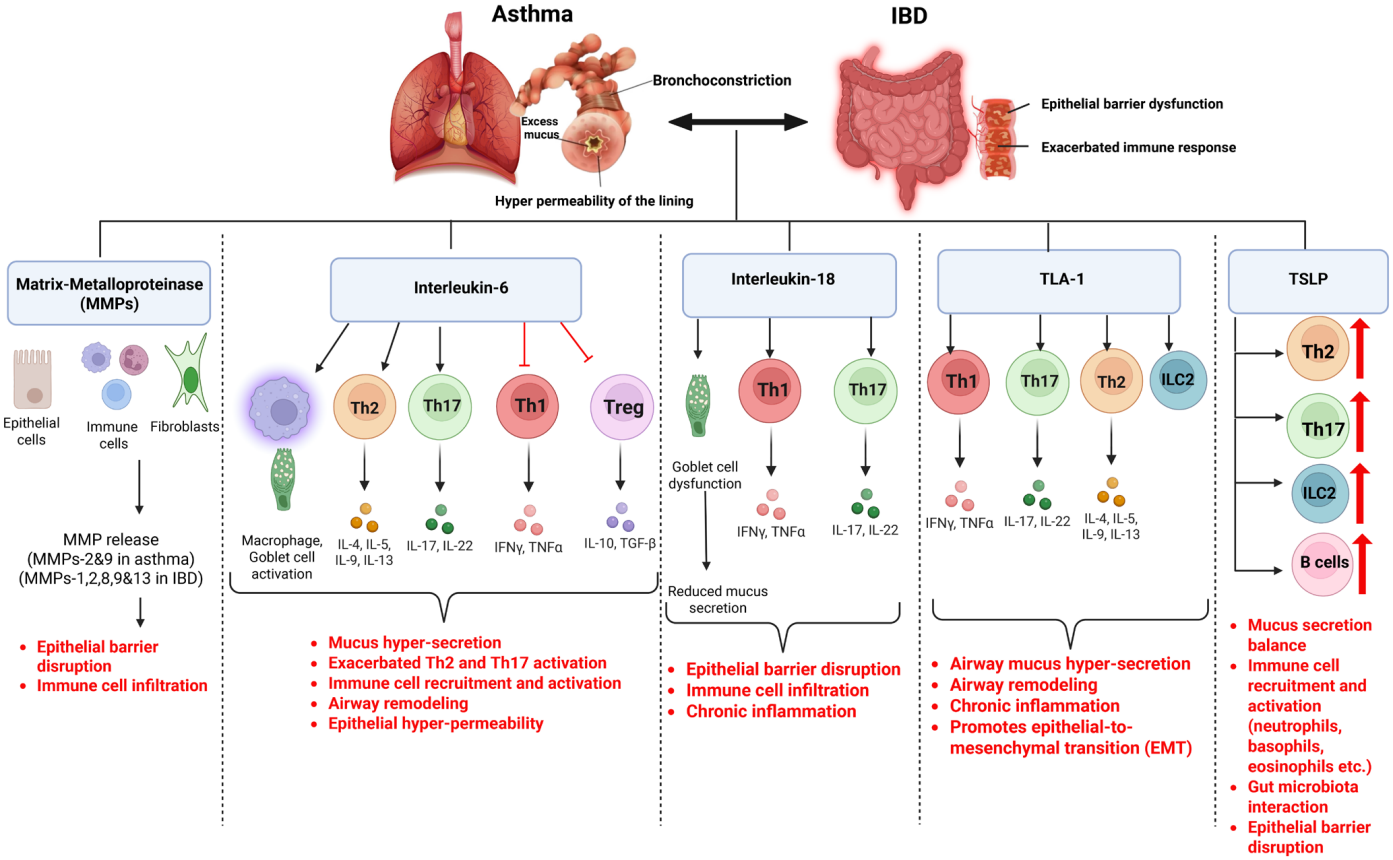
Schematic of inflammatory mediators potentially involved in the lung–gut axis and their detailed contributions to both asthma and IBD. Figure created with Biorender.com.

Epithelial barrier disruption caused due to excessive inflammation is the underlying cause of both asthma and UC. Several inflammatory mediators have been shown to be highly expressed in the setting of both asthma and IBD (Figure 2).

1. *Proteases*: Proteases play a significant role in both asthma and IBD, mainly in mediating epithelial barrier disruption leading to increased infiltration of leukocytes. Proteases act mainly via seven‐membrane G‐protein‐coupled receptors (GPCRs) and protease‐activated receptors (PARs) and are highly expressed in both asthma and IBD. Activation of PARs by proteases causes desquamation and the release of inflammatory cytokines, leading to degranulation of MCs and eosinophils, the main characteristic feature of asthma (Reed and Kita 2004). Similarly, in IBD, increased MMP or protease expression in inflamed colonic mucosa is associated with severe epithelial barrier dysfunction and increased extracellular matrix (ECM) remodeling, including collagen and elastin. While MMP‐2 and MMP‐9 are implicated in asthma (Kelly and Jarjour 2003; Takahashi et al. 2019), MMP‐9, MMP‐1, MMP‐13, MMP‐2, and MMP‐8 have been shown to be extensively involved in the progression of UC (Derkacz et al. 2021; Garg et al. 2009).
2. *Cytokines*: IL‐33 and IL‐13 are important interleukins involved in both asthma and IBD. IL‐33 is mainly produced by the damaged epithelium in the inflamed gut tissue, promoting the activation of Th2 and group 2 innate lymphoid cell (ILC2) immune responses and subsequent release of IL‐4, IL‐5, and IL‐13. It acts as an alarm to recruit more immune cells to the site of injury. IL‐33 overproduction in asthma leads to airway hyperresponsiveness, excessive mucosal deposition, and eosinophilia (Sjoberg et al. 2017). Interestingly, IL‐33 in UC has a dual role, both protective and inflammatory. Fibroblast‐secreted IL‐33 in the colonic mucosa has been shown to be essential for epithelial repair (Waddell et al. 2021), while dysregulated IL‐33 expression elevated mucosal inflammation, epithelial barrier disruption, and fibrosis. IL‐33 levels were found to be significantly upregulated in the colonic epithelial cells and infiltrating immune cells of the patients with active UC compared to healthy controls (Pastorelli et al. 2010). Eosinophilic activation, survival, and recruitment, which are commonly observed in asthma, are greatly mediated by IL‐13 action, and increased levels of IL‐13 mRNA were observed in the sputum and airway mucosa samples of patients with eosinophilic disorders. Additionally, IL‐13 protein levels increased in the blood and airway tissue (consisting of bronchial mucosa) of patients with eosinophilic disorders. IL‐13 was also shown to induce goblet cell metaplasia and increase mucus secretion and also cause increased airway hyperreactivity (Doran et al. 2017). Similarly, Fuss et al. investigated the effect of IL‐13 in UC and found significantly elevated levels of IL‐13 produced by the lamina propria T cells (LPT cells) in patients with UC compared to healthy individuals (Fuss et al. 2004).

IL‐6 and IL‐18 play complex roles in both asthma and UC, demonstrating the intricate immune mechanisms involved. IL‐6 is a major cytokine that is essential for producing IL‐4 during Th2 differentiation, promoting Th17 differentiation while inhibiting Th1 differentiation (Diehl and Rincon 2002). Since asthma is mainly characterized by Th2 inflammation, multiple studies have focused on identifying the role of IL‐6 in asthma. IL‐6 levels were increased in serum samples of asthmatic patients (Yokoyama et al. 1995) and in the bronchoalveolar lavage fluid (BALF) of active asthma patients when compared to healthy or stable asthma patients (Tillie‐Leblond et al. 1999). Although neutrophils and macrophages are the major secretors of IL‐6, some studies have shown increased levels of IL‐6 produced by airway epithelium (Marini et al. 1992; Neveu et al. 2011). Interestingly, IL‐6 in IBD has both protective and causal roles. Multiple studies have demonstrated the pathogenic effect of IL‐6 on the progression of UC (Cheng et al. 2024; Wu, Wei, et al. 2022). Although IL‐6 can lead to epithelial barrier dysfunction by upregulating claudin‐2 protein expression in intestinal epithelial cells, it is also found to be important for wound healing and epithelial cell survival (Alhendi and Naser 2023). Similar to IL‐6, IL‐18 is both pro‐ and anti‐inflammatory depending on the context. IL‐18 in severe asthma contributes to chronic airway inflammation, airway hyperresponsiveness (AHR), and mucus secretion (Thawanaphong et al. 2024). However, findings of Nowarski et al. demonstrated that IL‐18 equilibrium is crucial to maintaining epithelial barrier integrity and that IL‐18 deletion in intestinal epithelium led to an aggravated form of UC (Nowarski et al. 2015). Importantly, Zou et al. in 2023 established a causal relationship between asthma and UC through a two‐step, two‐sample MR study in which IL‐18 was found to contribute to a small extent (Zou et al. 2023).

Tumor necrosis factor (TNF)‐like factor 1 (TLA‐1) is a homotrimeric member of the TNF family that is activated either membrane‐bound on lamina propria or cleaved into soluble form by proteases, leading to excessive inflammation via activation of Th2 immune responses. Neutralizing TLA‐1 with antibodies decreased disease severity in an ovalbumin‐induced murine model of asthma (Fang et al. 2008). TLA‐1 is also majorly involved in the activation of ILC2, which plays a pivotal role in asthma development and progression (Meylan et al. 2014). Likewise, UC progression was alleviated when TLA‐1 was completely blocked in a DSS‐induced colitis model (Takedatsu et al. 2008). Antibody treatment against TLA‐1 improved symptoms of UC in mice (Meylan et al. 2011; Shih et al. 2014). Importantly, in experimental models, anti‐TLA1 antibodies are effective in the treatment of both UC and asthma (Clarke et al. 2018).

Thymic stromal lymphopoietin (TSLP) is yet another pro‐inflammatory mediator that is widely produced by epithelial cells, DCs, fibroblasts, basophils, and MCs. It mediates inflammation by the promotion of Th2 cell differentiation and activation, regulatory T cell differentiation, stimulation of IgA production by B cells (Park et al. 2017), promotion of eosinophilic inflammation (Ziegler and Artis 2010), activation of ILC2s (Kabata et al. 2020), and induction of Th17 responses (Tanaka et al. 2009). However, studies have implicated a protective role for TSLP in inflammatory diseases such as UC (Tahaghoghi‐Hajghorbani et al. 2019). This is because TSLP is majorly present on epithelial cells in two isoforms, a short form TSLP (sfTSLP) and a long form TSLP (lfTSLP). Findings of Fornasa et al. in 2015 detail the anti‐inflammatory effect of sfTSLP and the pro‐inflammatory effect of lfTSLP in IBD. While sfTSLP is released to the extracellular environment under homeostatic conditions to suppress the inflammatory cytokine release by DCs and peripheral blood mononuclear cells (PBMCs), lfTSLP is produced exclusively during inflammation (Fornasa et al. 2015). Therefore, while sfTSLP can be protective for IBD (Tahaghoghi‐Hajghorbani et al. 2019), lfTSLP can aggravate IBD severity (Zhuang and Li 2023). Similarly, multiple studies have shown a positive correlation between excessive lfTSLP production and asthma severity (Liang et al. 2022; Zhao et al. 2021). These findings highlight the complex and sometimes contradictory roles of cytokines in asthma and UC, emphasizing the need for a context‐specific understanding of their functions in the lung–gut axis.

iii *Leukotrienes*: LTs are pro‐inflammatory lipid mediators that are derived from arachidonic acid and have been extensively studied in the setting of allergy and asthma. Further, multiple studies have described their pathogenic role in IBD, especially in UC.

## 2 LT Biosynthesis and Receptors

**FIGURE 3:**
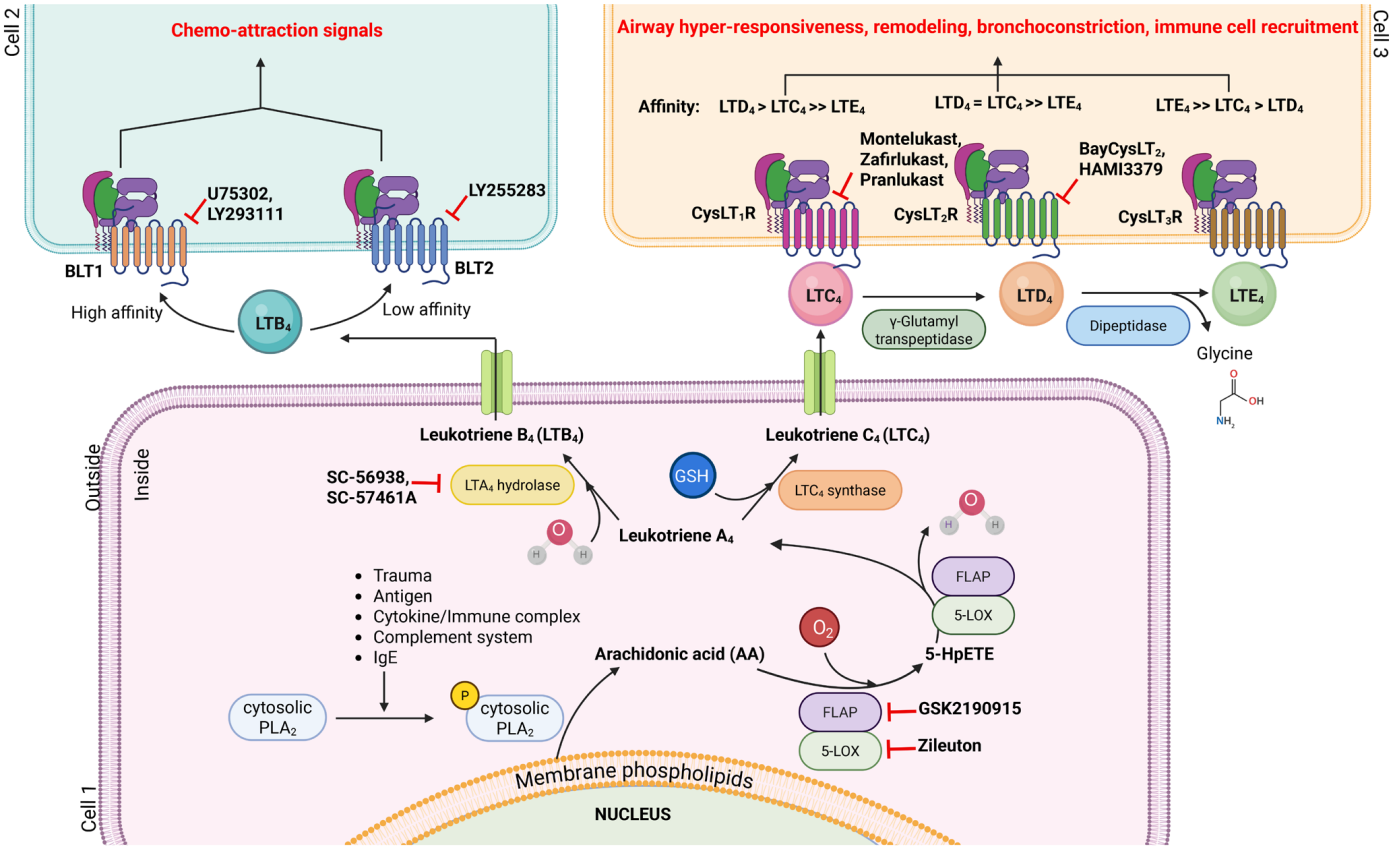
Leukotriene biosynthesis pathway. Various stimuli trigger the phosphorylation and activation of cytosolic phospholipase A_2_ (PLA_2_), which releases arachidonic acid (AA) from membrane phospholipids. The 5‐lipoxygenase enzyme (5‐LO) and its molecular partner, 5‐LOX activating protein (FLAP), convert AA to 5‐hydroperoxyeicosatetraenoic acid (5‐HpETE) and then to an unstable intermediate leukotriene A_4_ (LTA_4_). LTA_4_ is further converted to LTB_4_ by the LTA_4_ hydrolase enzyme. LTB_4_ acts via two receptors, BLT1 or BLT2, to exert its chemo‐attractive signals. LTA_4_ can also be converted to leukotriene C_4_ (LTC_4_) by its conjugation to glutathione via the action of LTC_4_ synthase. LTC_4_ is then extracellularly transported and sequentially converted to leukotriene D_4_ (LTD_4_) and leukotriene E_4_ (LTE_4_) by γ‐glutamyl transpeptidase and dipeptidase, respectively. LTC_4_, LTD_4_, and LTE_4_ act via three receptors, CysLT_1_R, CysLT_2_R, or CysLT_3_R (less common receptor for LTE_4_) to induce airway hyperresponsiveness, remodeling, bronchoconstriction, and immune cell recruitment. Figure created with Biorender.com.

LTs are derivatives of arachidonic acid generated by MCs, eosinophils, basophils, and macrophages at the site of injury (Kanaoka and Boyce 2004). Activation of each of these cell types due to trauma, antigen, immune complex, complement system, cytokines, or IgE results in the liberation of arachidonic acid from membrane phospholipids by a calcium‐dependent cytosolic phospholipase A_2_ (Clark et al. 1991) (Figure 3). The released arachidonic acid is further metabolized by the action of either the cyclooxygenase enzyme (COX) or the 5‐lipooxygenase enzyme (5‐LO). While the cyclooxygenase enzyme pathway produces prostaglandins and thromboxane, the 5‐lipoxygenase enzyme is the sole producer of the leukotriene pathway. Arachidonic acid is then converted to the unstable intermediate leukotriene A_4_ (LTA_4_) by the actions of the 5‐lipoxygenase (5‐LO) enzyme and its molecular partner, 5‐LO activating protein (FLAP), at the nuclear envelope (Dixon et al. 1990; Malaviya et al. 1993). Subsequently, the LTA_4_ hydrolase enzyme converts LTA_4_ to produce leukotriene B_4_ (LTB_4_), a potent chemoattractant. On the other arm, LTA_4_ is conjugated to reduced glutathione by an integral nuclear membrane protein, leukotriene C_4_ synthase (LTC_4_S) (Lam et al. 1994; Nicholson et al. 1993), forming leukotriene C_4_ (LTC_4_). After active transport to the extracellular space by multidrug resistance protein (MRP)‐1 (Leier et al. 1994), LTC_4_ is converted extracellularly to leukotriene D_4_ (LTD_4_) by a γ‐glutamyl leukotrienase (Carter et al. 1998), and then to the terminal product leukotriene E_4_ (LTE_4_) by a dipeptidase (Lee et al. 1983). LTC_4_ and LTD_4_ are potent biomolecules, and their rapid conversion to LTE_4_ ensures that they are very short‐lived in vivo. In contrast, LTE_4_ is a stable molecule detected in biologic fluids and excreted in the urine without further modification (Drazen et al. 1992). LTC_4_, LTD_4_, and LTE_4_ are collectively called cysteinyl leukotrienes (cys‐LTs), as all three contain cysteine group in their structure (Jo‐Watanabe et al. 2019). Although cys‐LTs are mainly secreted by infiltrating LTC_4_S‐containing immune cells (Colazzo et al. 2017), endothelial cells (EC) can take up LTA_4_ released by leukocytes and generate cys‐LTs using microsomal glutathione S transferase‐2 and gamma glutamyl transpeptidases (Nicosia et al. 2001). While LTB_4_ is a potent chemoattractant for neutrophils (He et al. 2020), cys‐LTs play pivotal role in bronchoconstriction, exacerbated vascular permeability, chronic inflammation, eosinophilic recruitment, and MC degranulation, which are characteristic features of allergy and asthma. LTB_4_ acts via two GPCRs, BLT1 and BLT2 (Yokomizo et al. 1997, 2000). The main distinctions between BLT1 and BLT2 involve in their affinity and specificity for LTB_4_, along with their cellular distribution. BLT1 is the high‐affinity ligand for LTB_4_ and is most commonly expressed by many immune cells whereas certain epithelial cells also express BLT1 at lower levels. BLT2 on the other hand is the low‐affinity receptor for LTB_4_ and is predominantly expressed in epithelial cells in the gut and the skin. Apart from BLT1 and BLT2 receptors, LTB_4_ can also interact with nuclear receptor peroxisome proliferator‐activated receptor α (PPARα) (Devchand et al. 1996). Additionally, studies by Okuno et al. and Liu et al. demonstrated the ability of the BLT2 receptor to respond to other molecules such as 12‐hydroxyheptadecatrienoic acid apart from LTB_4_ (Liu et al. 2014; Okuno et al. 2008). Cys‐LTs are recognized by at least two known GPCRs, respectively termed cysteinyl leukotriene receptor 1 (CysLT_1_R) and cysteinyl leukotriene receptor 2 (CysLT_2_R) and mediate their biologic functions (Heise et al. 2000; Lynch et al. 1999). CysLT_1_R is expressed prominently by smooth muscle cells and by several leukocytes (Lynch et al. 1999; Figueroa et al. 2003), while CysLT_2_R is expressed by cardiac Purkinje cells, endothelial cells (EC), brain, and leukocytes (Heise et al. 2000). A recent study from our lab demonstrated that CysLT_2_R in EC promotes angiogenesis and vascular permeability in a murine model of lung cancer (Duah et al. 2019). CysLT_1_R binds LTD_4_ with higher affinity than LTC_4_ (EC50 for binding of 10^−9^ and 10^−8^ M, respectively) (Lynch et al. 1999), while CysLT_2_R has equal affinity for LTD_4_ and LTC_4_ (EC50 of 10^−8^ M for each) (Heise et al. 2000). LTE_4_ is only a weak, partial agonist for CysLT_1_R and CysLT_2_R, binding each at 1–2‐log fold lower affinity than LTC_4_ and LTD_4_ (Heise et al. 2000; Figueroa et al. 2003). Another receptor, termed GPR17, recognizes both LTD_4_ and uracil nucleotides and is expressed primarily in the brain (Ciana et al. 2006) and negatively regulates CysLT_1_R (Maekawa et al. 2009). LTE_4_, despite being a weak agonist for CysLT_1_R, exhibits unique properties. It induces pulmonary responses in vivo that are not recapitulated by LTC_4_ or LTD_4_. Importantly, these responses cannot be fully explained by the known pharmacology of established GPCRs for cys‐LTs. We have shown that LTE_4_ markedly exceeds the potency of LTD_4_ for inducing proliferation, ERK activation, chemokine generation, and COX‐2 expression in MCs via peroxisome proliferator‐activated receptor gamma (PPARγ) (Paruchuri et al. 2008). We also demonstrated that the P2Y_12_ receptor (P2Y_12_R) relays LTE_4_ signals in activating MC and amplifying mucosal inflammation induced by low‐dose allergen in sensitized BALB/c mice (Paruchuri et al. 2009). However, we could not detect any direct binding of LTE_4_ to P2Y_12_R. Recently, GPR99 has been identified as a potential third CysLTR with a preference for LTE_4_ (Kanaoka et al. 2013) and relays LTE_4_‐induced mucin release (Bankova et al. 2016) and is important for the production of IL‐25 by the airway brush cells (Bankova et al. 2018).

## 3 LTs in Asthma

**FIGURE 4:**
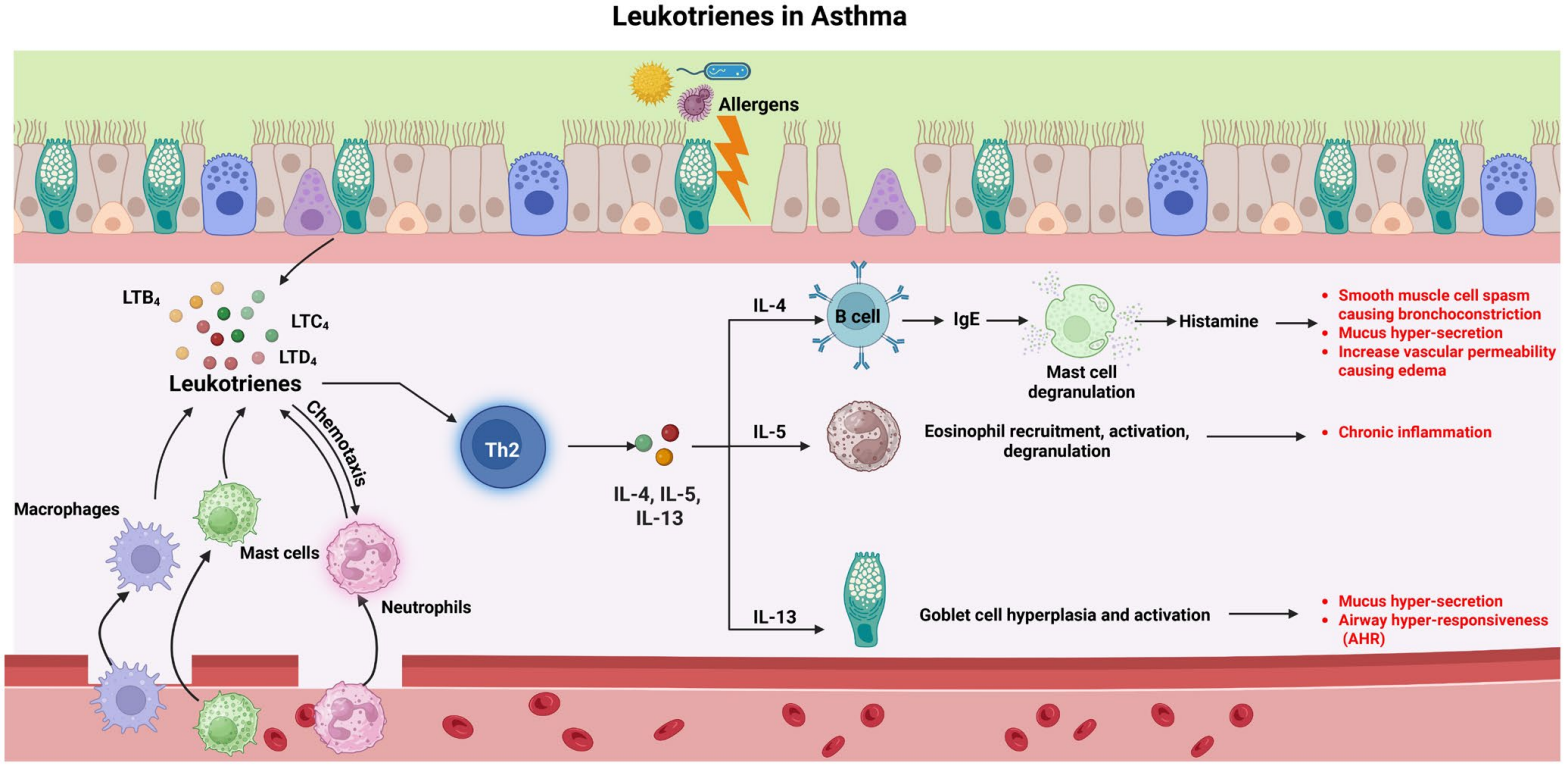
Overview of the role of leukotrienes (LTs) in asthma: Allergen exposure can trigger the production of LTs from different cell types in the airway. LTB_4_ acts as a chemoattractant for neutrophils and other immune cells such as macrophages and MCs, which rush to the site of injury and get activated, generating LTs. LTs mainly mediate the activation of Th2‐type immune response, which leads to the production of different inflammatory cytokines such as IL‐4, IL‐5, and IL‐13. IL‐4 mediates B cell activation, which in turn produces IgE antibodies. During the challenge phase, IgE binding to MCs causes degranulation and release of histamine, which contributes to asthma manifestations. IL‐5 mediates eosinophilic recruitment, activation, and degranulation, which contributes to further chronic inflammation. IL‐13 is essential to activate goblet cells to release mucus, resulting in airway hyperresponsiveness observed during asthma. Figure created with Biorender.com.

Numerous studies have investigated the effect of LTs in asthma induction and progression, finding a positive correlation. Figure 4 summarizes the broad role of LTs in the pathogenesis of asthma. Higher protein levels of 5‐LO and LTA_4_ hydrolase enzymes were observed in the bronchial biopsies of asthma patients compared to healthy controls through immunohistochemistry, whereas increased levels of 5‐LO and LTA_4_ hydrolase transcripts were detected in peripheral polymorphonuclear leukocytes from asthmatic children compared to healthy controls (Seymour et al. 2001; Zaitsu et al. 2003). Additionally, elevated levels of LTB_4_ were observed in the BALF, blood, and exhaled breath condensate (EBC) in children and adolescents suffering from asthma (Trischler et al. 2015). Sputum samples from patients with bronchial asthma revealed the presence of LTs (O'Driscoll et al. 1984). Several studies have shown that LTB_4_ is essential for T cell activation, particularly for the transmigration of CD8^+^ T cells to lungs and for the release of IL‐13 (Arcoleo et al. 1995; Farrar and Humes 1985; Miyahara et al. 2005; Rola‐Pleszczynski et al. 1986). Although LTB_4_ is important in asthma pathogenesis, more extensive research has focused on cys‐LTs. Traces of LTE_4_, the stable product of cys‐LTs, were observed in the urine samples of asthmatics and serve as a biomarker for asthma (Hoffman and Rabinovitch 2018). As a class, cys‐LTs are the most potent known direct bronchoconstrictors (Davidson et al. 1987; Drazen and Austen 1987), and also potentiate AHR to histamine when administered by inhalation to human subjects (Christie et al. 1992). Multiple studies have provided evidence for cys‐LTs being 10,000 times more potent bronchoconstrictors than histamine and methacholine (Adelroth et al. 1986; Drazen et al. 1980; Weiss et al. 1982). Cys‐LTs are also responsible for airway remodeling and increased vascular permeability. Endobronchial challenge of atopic asthmatic subjects with LTE_4_ elicits significant increases in sputum eosinophils, basophils, and MCs at 7 and 24 h (Gauvreau et al. 2001). Instillation of LTE_4_ also increases the number of eosinophils in bronchial biopsies obtained from asthmatic subjects 4 h after the challenge (Laitinen et al. 1993). Subjects with aspirin‐exacerbated respiratory disease (AERD), a variant of asthma characterized by enhanced generation of cys‐LT proteins in the airway, exhibit bronchoconstrictor responses to inhaled LTE_4_ (Arm et al. 1990). Prior inhalation of LTE_4_ by asthmatic subjects potentiates AHR to histamine; this can be blocked by pre‐treatment of the subjects with the cyclooxygenase (COX) inhibitor, indomethacin (O'Hickey et al. 1991). Similarly, LTE_4_ potentiates the contraction of guinea pig tracheal rings to histamine in an indomethacin‐sensitive fashion (Lee et al. 1984). The above studies suggest that prostanoids may mediate some of the unique actions of cys‐LTs. Along these lines, we identified a novel cys‐LT‐prostaglandin E_2_ crosstalk, where LTD_4_ shifts PGE_2_ signaling to the EP_3_R/PKG axis in MC, potentiating inflammation (Kondeti et al. 2016). Cys‐LTs also contribute to airway remodeling in chronic asthma by promoting the proliferation of airway smooth muscle and epithelial cells, as well as enhancing collagen deposition (Panettieri Jr. 1998). Moreover, cys‐LTs contribute to the inflammatory aspects of asthma by promoting microvascular permeability. We have recently shown that cys‐LTs via CysLT_2_R in EC were able to induce microvascular hyperpermeability by promoting endothelial cell contraction leading to increased vascular leakage (Duah et al. 2013, 2019). We also demonstrated that cys‐LTs potentiate SCF‐mediated MC hyperplasia and could culminate in hyperreactivity observed during airway hyperresponsiveness (Al‐Azzam et al. 2015). These findings, together with many others, clearly indicate the crucial role of LTs in the development and progression of asthma. LTs, including LTB_4_ and cys‐LTs, exert their pathogenic effect in asthma via each of their receptors expressed differentially on various cell types involved in the process (Hallstrand and Henderson Jr. 2010). For example, cys‐LTs act via CysLT_1_R on immune cells and bronchial mucosa to promote bronchoconstriction, vascular permeability, and mucus secretion. Cys‐LTs can also act via the CysLT_2_R on smooth muscle cells and EC to induce fibrosis and tissue remodeling. On the other hand, LTB_4_ can act via BLT1 expressed by immune cells such as T cells to recruit neutrophils to the site of injury. Whereas LTB_4_ can also bind to the BLT2 receptor on airway smooth muscle cells to promote inflammation. The mechanisms that control cys‐LT‐dependent biologic responses are thus of considerable pathobiological and clinical interest in both allergic and nonallergic diseases.

## 4 LTs in IBD

**FIGURE 5:**
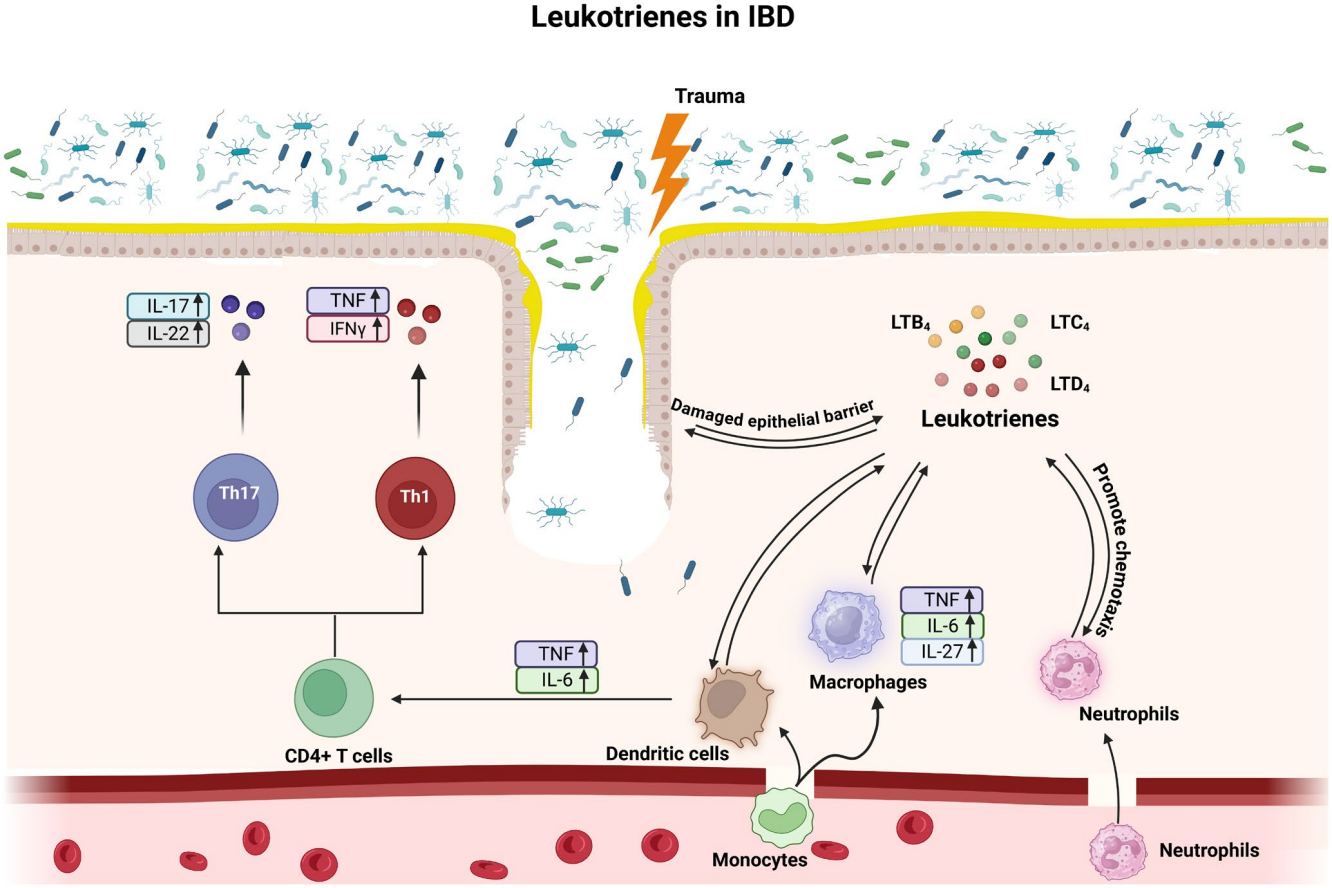
Overview of the role of leukotrienes (LTs) in IBD: LTs produced as a result of damaged epithelial barrier due to various traumatic events can contribute to enhanced epithelial barrier dysfunction, recruitment and activation of various immune cell components such as macrophages, DCs, and neutrophils. While neutrophils can promote further acute inflammation, macrophages release several pro‐inflammatory mediators such as tumor necrosis factor (TNF), IL‐6, and IL‐27. Elevated levels of TNF and IL‐6 from DCs can activate and differentiate CD4^+^ T cells into Th17 and Th1 lineages, thereby producing even more TNF, interferon gamma (IFNγ), IL‐17, and IL‐22. This enhanced release of cytokines potentially leads to chronic inflammation observed in IBD. Figure created with Biorender.com.

LTB_4_ levels were shown to be elevated in the serum samples of patients with Crohn's disease, indicating the involvement of the 5‐LO pathway in IBD pathogenesis (Kikut et al. 2022). LTB_4_ was shown to be secreted by the colonic mucosal layer in IBD patients (Sharon and Stenson 1984). This study suggests that LTB_4_ acts as a potent chemoattractant, recruiting immune cells to the colon and initiating chronic inflammation. Additionally, investigation of resected mucosa obtained from 7 control patients and 10 patients with UC revealed increased LTB_4_ activity and higher neutrophil recruitment in UC patients compared to controls (Cole et al. 1996). A study comparing the levels of LTB_4_ and prostaglandin secretion by the mucosa in the ileal pouch of UC patients and familial adenomatous polyposis (FAP) patients discovered significantly greater LTB_4_ production in UC patients than FAP patients (Gertner et al. 1994). Another interesting study demonstrated that smoking reduced the levels of LTB_4_ which were significantly higher in the colonic mucosa tissue sample of UC patients compared to healthy controls (Motley et al. 1990). Similar to asthma, LTB_4_ exerts its effects via both BLT1 and BLT2 receptors in UC. While BLT1 is shown to have detrimental effects in colitis by promoting neutrophil recruitment and subsequent inflammation, BLT2 expressed in colon cryptic cells appears to protect mice against DSS‐induced colitis, possibly by enhancing barrier function in epithelial cells of the colon (Iizuka et al. 2010). Along the same lines, significantly higher levels of cys‐LTs were produced by the rectal mucosal layer in both UC and Crohn's disease patients compared to healthy controls (Peskar et al. 1986). Importantly, urinary LTE_4_ levels were higher in active IBD patients compared to those in remission (Stanke‐Labesque et al. 2008), suggesting that urinary LTE_4_ could be an interesting non‐invasive biomarker for IBD. Hammerbeck and Brown in 1996 reported an increased cys‐LT expression in the inflamed distal colon of guinea pigs (Hammerbeck and Brown 1996). Further, multidrug resistance‐associated protein 1 (MRP1), which is the major transporter of LTC_4_, was shown to be highly expressed in inflamed epithelium of UC and Crohn's disease patients (Blokzijl et al. 2008). Notably, pharmacological inhibition of leukotriene synthesis was shown to improve colonic ulcers and colitis resolution in preclinical models (Wallace et al. 1989). Both CysLT_1_R and CysLT_2_R have a negative impact on IBD. Pharmacological inhibition of CysLT_1_R using Montelukast in an acetic acid‐induced model of colitis in rats led to reduced colonic damage and disease severity (Ghorbanzadeh et al. 2022). Similarly, CysLT_2_R knockout mice exhibited better protection from DSS‐induced colitis, less colonic edema (potentially via less venule permeability), and attenuated TNF‐α production compared to WT mice (Barajas‐Espinosa et al. 2011). Figure 5 summarizes the broad role of LTs in the pathogenesis of IBD.

Since gut microbiota has a crucial role in the pathogenesis of IBD, including UC, the effect of gut microbiota on leukotriene synthesis and signaling pathways further supports the strong association between LTs and IBD. As previously stated, LPS from gut bacteria can activate TLR4, leading to the production of IL‐13 by MCs via the MyD88‐BLT2 pathway (Lee et al. 2017). There is significant protein upregulation of the BLT2 receptor and its ligands in LPS‐induced bone‐marrow‐derived MCs during this process, depicting the effect of the gut microbiome in the leukotriene pathway in immune cells. Additionally, Doré et al. demonstrated a possible interaction between systemically secreted phospholipase A_2_‐IIA (a key anti‐microbial peptide) and the gut microbiome leading to increased inflammation (Dore et al. 2022). Furthermore, gut microbial species such as *Lactobacillus plantarum* can metabolize polyunsaturated fatty acids (PUFA) like arachidonic acid (the parent substrate of the leukotriene pathway) into saturated fatty acids that could potentially limit the substrate availability for leukotriene synthesis (Kishino et al. 2013). Another possible mechanism through which the gut microbiota can influence leukotriene synthesis could be through their secondary metabolites such as SCFAs. As previously discussed, SCFAs have been shown to exert epigenetic modifications, and in agreement, SCFAs, especially butyrate, exhibited an inhibitory effect on lipoxygenase mRNA expression in intestinal monolayer cells (Ohata et al. 2005). The strong positive correlation between LTs and the development and progression of both asthma and IBD, particularly UC, suggests a crucial role for LTs in the lung–gut axis. The following section of this review will explore treatment strategies targeting leukotriene signaling to address both asthma and IBD treatments more effectively.

## 5 LT Antagonists in the Treatment of Asthma and IBD

Blocking the leukotriene pathway hierarchically begins at the 5‐LO level, which is the foremost step in the production of LTB_4_ and cys‐LTs. Zileuton is the most common inhibitor of the 5‐LO enzyme that works by chelating the iron site of the 5‐LO. Zileuton has been shown to reduce the production of LTs in the airway and thus reduce asthma severity in clinical trials (Liu et al. 1996). Another method to block the effects of 5‐LO is by targeting FLAP. GSK2190915 was identified as a FLAP inhibitor and was successful in reducing LTB_4_ and urinary LTE_4_ levels in asthma patients. However, it was unsuccessful in limiting neutrophil infiltration and disease severity (Chaudhuri et al. 2014). Since blocking 5‐LO or FLAP can also inhibit the production of other anti‐inflammatory mediators like resolvins and lipoxins, inhibiting candidates from the level below LTA_4_ hydrolase or LTC_4_S would be ideal in addressing asthma and other chronic inflammatory diseases like IBD. Several inhibitors of LTA_4_ hydrolase have been developed in the past decade for multiple disease conditions, including asthma. However, only a few of them entered phase 2 clinical trials, and just one among them reached the market under the brand name ubenimex (bestatin) for the treatment of pulmonary arterial hypertension (PAH) and lymphedema (Bhatt et al. 2017). Although shown to reduce LTA_4_ hydrolase enzyme activity, these drugs are not effective in treating asthma. Recent efforts have focused on designing inhibitors that selectively target the epoxide hydrolase activity of LTA_4_ hydrolase while sparing the aminopeptidase function. Examples include SC‐56938, SC‐57461A (Bhatt et al. 2017; Rohn et al. 2021). Yet another method to inhibit LTB_4_ signaling is by using antagonists of the BLT receptors. LY293111 and U75302 are the two most common BLT1 antagonists that have also been shown to successfully inhibit LTB_4_ signaling by competing for the BLT1 receptor. U75302 has been shown to alleviate LPS‐mediated cardiac dysfunction by inhibiting the LTB_4_/BLT1 signaling (Sun et al. 2017) whereas the action of LY293111 is more studied in pancreatic and colon cancer models (Hennig et al. 2004, 2005). Apart from BLT receptor antagonists, certain herbal therapies have also shown to reduce LTB_4_ levels, especially in IBD patients. Nicotine derived from *Nicotiana tabacum* has been demonstrated to decrease the levels of LTB_4_ in colitis induced by dinitrobenzene‐sulfonic acid (Ko and Cho 2005).

Targeting cys‐LT signaling, on the other hand, is primarily achieved through CysLT_1_R and CysLT_2_R antagonists. Zafirlukast, an indole derivative, was the first CysLT_1_R antagonist to be FDA‐approved in 1996 for the treatment and management of chronic asthma in patients above five years of age. Montelukast, a quinoline derivative, was FDA‐approved 2 years later in 1998 for the treatment of mild to moderate asthma in six‐year‐old and above patients (Valacer 1999). Pranlukast, approved in 2007, is yet another CysLT_1_R antagonist that is widely used in countries, including Japan, Mexico, Argentina, and Colombia. While oral bioavailability of Zafirlukast is about 80%, montelukast has only 65% oral availability. CysLT_1_R antagonists have been shown to be highly effective in many disease conditions apart from asthma (Biagioli et al. 2023; Wu, Cui, et al. 2022; Zovko et al. 2018). Zafirlukast is effective against the toxic effects of acetic acid in the colon of the rats (Mahgoub et al. 2003). Although CysLT_1_R antagonists like Zafirlukast and Montelukast are FDA‐approved for patients, CysLT_2_R antagonists including HAMI3379 and BayCysLT_2_, remain unapproved for asthma treatment. Apart from CysLT_2_R, HAMI3379 also blocks GPR17. We and others have shown that both HAMI3379 and BayCysLT_2_ effectively inhibit CysLT_2_R, reducing disease severity in experimental models (Duah et al. 2019; Barajas‐Espinosa et al. 2012; Chen et al. 2024; Lin et al. 2014; Merten et al. 2018; Ni et al. 2011; Sekioka et al. 2017; Zhou et al. 2022). However, limited clinical trials have been performed on CysLT_2_R antagonists to determine the efficacy and safety of the drug. A new drug called Gemilukast (also known as ONO‐6950), which antagonizes both CysLT_1_R and CysLT_2_R through its dual action, is currently under FDA review for approval. Clinical trials led by Gauvreau et al. witnessed a significant reduction in airway inflammation with the use of ONO‐6950 (Gauvreau et al. 2016). Table 2 summarizes the above list of leukotriene pathway inhibitors developed for various pathological conditions. Since LTs have been shown to be important for the development and progression of UC, employing well‐tested leukotriene pathway antagonists may help improve disease prognosis. However, further studies and clinical trials are required to fully investigate the effects of CysLTR antagonists in treating IBD.

**TABLE 2:** List of some of the leukotriene pathway antagonists/inhibitors developed for various diseases.

Inhibitor/antagonist	Target	Disease	FDA‐approval
Zileuton (Zyflo) (Liu et al. 1996)	5‐LO	Asthma	Yes (1996)
GSK2190915 (Chaudhuri et al. 2014)	FLAP	Asthma	No
Bestatin (Ubenimex) (Bhatt et al. 2017)	LTA_4_ hydrolase	Pulmonary arterial hypertension (PAH), Acute myeloid leukemia	Granted orphan drug designation
Zafirlukast (Accolate) (Valacer 1999; Mahgoub et al. 2003)	CysLT_1_R	Chronic asthma	Yes (1996)
SC‐57461A (Rohn et al. 2021)	LTA_4_ hydrolase	Asthma	No
LY293111 (Etalocib) (Hennig et al. 2004, 2005)	BLT1 receptor	Non‐small cell lung cancer (NSCLC), Pancreatic cancer	No
Pranlukast (Valacer 1999; Biagioli et al. 2023)	CysLT_1_R	Asthma	No, but widely used in other countries including Japan, Mexico, Argentina, and Columbia
Montelukast (Valacer 1999; Wu, Cui, et al. 2022; Zovko et al. 2018)	CysLT_1_R	Mild to moderate asthma	Yes (1998)
HAMI3379 (Chen et al. 2024; Lin et al. 2014; Merten et al. 2018; Zhou et al. 2022)	CysLT_2_R	Ischemic brain injury, Asthma	No
BayCysLT_2_ (Duah et al. 2019; Ni et al. 2011)	CysLT_2_R	Coronary artery disease, COPD	No
Gemilukast (ONO‐6950) (Gauvreau et al. 2016)	Both CysLT_1_R and CysLT_2_R	Asthma	No

## 6 Animal Models to Study Asthma and UC

Ovalbumin and house dust mite (HDM)‐induced asthma models are the most commonly used murine models to study asthma (Bates et al. 2009). In the ovalbumin model, mice are sensitized initially with intraperitoneal injections of foreign protein such as ovalbumin along with adjuvants such as aluminum hydroxide. Following sensitization, multiple challenges of the same protein via aerosolization are done to induce AHR and eosinophilic recruitment. This model primarily induces eosinophilic inflammation (Th2‐driven), characterized by IL‐4, IL‐5, and IgE production. While ovalbumin induces acute asthma, the fact that it is an artificial allergen not naturally encountered by humans and requires the use of an adjuvant makes this model less preferred to employing a naturally occurring human allergen, HDM intranasally, which is linked to the majority of asthma cases (Bates et al. 2009). The HDM model better replicates chronic asthma features, including airway remodeling (e.g., mucus hyperplasia, collagen deposition) and persistent inflammation, due to repeated natural allergen exposure (Bates et al. 2009).

Animal models to study UC can be broadly classified into (i) chemically induced model, (ii) adoptive T‐cell transfer model, and (iii) genetically engineered model (Baydi et al. 2021). The chemically induced model mainly consists of the sodium dextran sulfate (DSS) model, 2,4,6‐trinitrobenzenesulfonic acid (TNBS)‐induced model, and the oxazolone‐induced model. Of the three, the DSS‐induced colitis model is the most widely used experimental model to study UC. This model involves administration of mice with DSS in drinking water for a period of 8–10 days to induce colonic epithelial barrier disruption and ultimately lead to colitis (Baydi et al. 2021). In the adoptive T‐cell transfer model, CD4^+^CD45RB^high^ T cells (naïve T cells) are isolated from healthy wild‐type mice and transferred intraperitoneally to SCID or RAG^−/−^ mice (immunodeficient). Aberrant activation of T cells by gut microbiota leads to chronic inflammation over a span of 4–8 weeks mediated via the Th1/Th17 axis. The adoptive T‐cell transfer model allows researchers to study the early stages of colitis and gut inflammation, making the model more relevant to human IBD. Genetically engineered animal models employ IL‐10 global knockout mice as IL‐10 being an anti‐inflammatory cytokine, has a beneficial effect on colitis. Deletion of IL‐10 causes spontaneous colitis in mice after 3 months of age. The infiltrate in the colon constitutes high levels of lymphocytes, neutrophils, and macrophages, indicating colonic inflammation and subsequent colitis (Baydi et al. 2021).

Although several animal models are available to study the pathogenesis of asthma and IBD individually, currently, there are no animal models available to study the link between asthma and IBD. An ideal model would be the one in which the induction of a single disease condition is sufficient to trigger the development of the other disease condition without the need for any external stimuli. For example, by inducing asthma through one of the above‐mentioned methods and then studying the effects on the colon, or by inducing colitis and then determining its effect on the lungs. Employing these models could elucidate the role of LTs in the asthma–IBD axis.

## 7 Neuronal Impact on the Lung–Gut Axis

Studies linking the brain, lungs, and gut have rapidly expanded over the past 5 years. A recent study highlights the importance of neuronal signals in driving the gut–lung axis. The study provides evidence that feeding activates the parasympathetic nervous system, leading to the stimulation of ILC2 cells via the acetylcholine‐cholinergic receptor muscarinic 4 (Chrm4) axis causing an ILC2‐mediated type‐2 allergic response in mice (Chen et al. 2025). Alternately, signals from lungs were also shown to impact the gut through the nervous system (Wang et al. 2024). Following respiratory syncytial virus (RSV) infection, IL‐1α protein levels were increased in the brain, causing regulation of appetite‐associated genes like leptin and weight loss and altering the gut microbiota. Notably, inhibition of IL‐1α reversed the changes in gut microbiota commonly observed after RSV infection (Wang et al. 2024).

However, the lung–gut–brain axis is a fairly new area in research with a limited amount of evidence supporting the direct involvement of the nervous system in the lung–gut axis. Importantly, it is unclear if the crosstalk is linear from one organ to another or rather triaxial. In a model of pulmonary hypertension (PH), the signals from the lung led to altered gut microbiota and gut wall leakiness associated with activation of microglia and presence of neuroinflammation in the brain regions (Sharma et al. 2020). Likewise, zinc oxide nanoparticle inhalation in the lungs was shown to affect both the gut and the brain by altering the gut microbiota and promoting cortex damage (Zhang et al. 2023). Further, antibiotic depletion of gut microbiota in mice alleviated this cortex damage. Along similar lines, inhalation of fine particulate matter (PM_2.5_), a key pollutant, caused increased inflammatory cytokines in the BAL, followed by increased microbial species in the gut associated with neurodegeneration (Song et al. 2025). Several mechanisms were proposed to mediate the lung–brain axis or the gut–brain axis, although it is still unclear regarding the lung–brain–gut axis. The lung–brain axis has been shown to be mediated via the hypothalamic–pituitary–adrenal (HPA) axis, vagus nerve interaction, lung‐resident microbial metabolites, immune signaling primed by the lungs, and a state of hypoxia induced by chronic pulmonary complications (Li et al. 2023). During the events of stress (as in asthma), the HPA axis can get activated, leading to the increased production of glucocorticoids such as cortisol, which can suppress anti‐inflammatory effects on the Th2‐type immune reaction. Vagus nerve interactions are another factor connecting the lungs and the brain. The autonomic functions of the lungs, such as cough responses and alveolar tension, are relayed to the brainstem nuclei via the vagal afferent fibers, while the efferent fibers control the airway contraction and mucus secretion. Of note, immune cells such as ILCs in the lungs have been shown to express receptors for neurotransmitters such as acetylcholine. In addition, the microbial population residing in the lung can release metabolites such as LPS systemically, which have then been shown to cross the blood–brain barrier to increase hippocampal TNF‐α and amyloid‐beta accumulation. Similarly, the gut–brain axis has also been well‐characterized. Common mechanisms underlying the gut–brain axis involve the vagus nerve interaction, HPA axis dysregulation, gut microbial metabolite effects (SCFA and neurotransmitters), and immune signaling mediated by the gut (Kasarello et al. 2023; Zheng et al. 2023). In addition to these, the enteric nervous system (nerve cells within the GI tract), often referred to as the “second brain,” also plays a crucial role in the gut–brain axis. It is possible that similar mechanisms regulate the lung–brain–gut axis. However, further studies are required to validate the mechanisms regulating this axis.

## 8 Conclusions and Future Directions

This review concludes by suggesting that LTs could potentially be one of the mediators of the lung–gut axis. Detailed examination of the leukotriene biosynthesis pathway and their implications in asthma and IBD individually have highlighted the significance of targeting this signaling axis to enhance disease outcomes in patients with both asthma and IBD. Beyond LTs, this review has also contributed to a deeper understanding of the lung–gut axis, its various modulators, and different animal models to study asthma and UC, along with a brief discussion on the neuronal regulation of the lung–gut axis. For future prospects, animal models to study both asthma and UC simultaneously can be used to decipher the precise connecting links between the two, along with evaluating the role of LTs in this crosstalk. Additionally, employing antagonists targeting the leukotriene signaling pathway can be used on animal models of asthma and UC co‐manifestation to gauge the feasibility of utilizing these blockers by patients experiencing both asthma and UC. Furthermore, identifying the mechanistic aspects of the lung–gut–brain axis in the context of asthma and UC could offer novel neuromodulators to target both asthma and UC with better efficacy.

## Author Contributions

Emma Elizabeth Sabu Kattuman and Sailaja Paruchuri conceived the project, wrote the manuscript, and made the figures. Emma Elizabeth Sabu Kattuman, Lakshminarayan Reddy Teegala, Somayeh Darzi, Charles K. Thodeti, and Sailaja Paruchuri revised and edited the manuscript.

## Conflicts of Interest

The authors declare no conflicts of interest.

## Data Availability

The authors have nothing to report.

## References

[cph470022-bib-0001] Actis, G. C. , and F. Rosina . 2013. “Inflammatory Bowel Disease: An Archetype Disorder of Outer Environment Sensor Systems.” World Journal of Gastrointestinal Pharmacology and Therapeutics 4, no. 3: 41–46. 10.4292/wjgpt.v4.i3.41. 23919214 PMC3729865

[cph470022-bib-0002] Adelroth, E. , M. M. Morris , F. E. Hargreave , and P. M. O'Byrne . 1986. “Airway Responsiveness to Leukotrienes C4 and D4 and to Methacholine in Patients With Asthma and Normal Controls.” New England Journal of Medicine 315, no. 8: 480–484. 10.1056/NEJM198608213150803. 3526153

[cph470022-bib-0003] Al‐Azzam, N. , V. Kondeti , E. Duah , F. Gombedza , C. K. Thodeti , and S. Paruchuri . 2015. “Modulation of Mast Cell Proliferative and Inflammatory Responses by Leukotriene d4 and Stem Cell Factor Signaling Interactions.” Journal of Cellular Physiology 230, no. 3: 595–602. 25161061 10.1002/jcp.24777 PMC4244248

[cph470022-bib-0004] Alhendi, A. , and S. A. Naser . 2023. “The Dual Role of Interleukin‐6 in Crohn's Disease Pathophysiology.” Frontiers in Immunology 14: 1295230. 10.3389/fimmu.2023.1295230. 38106420 PMC10722226

[cph470022-bib-0005] Anand, S. , and S. S. Mande . 2018. “Diet, Microbiota and Gut‐Lung Connection.” Frontiers in Microbiology 9: 2147. 10.3389/fmicb.2018.02147. 30283410 PMC6156521

[cph470022-bib-0006] Ananthakrishnan, A. N. , E. L. McGinley , D. G. Binion , and K. Saeian . 2011. “Ambient Air Pollution Correlates With Hospitalizations for Inflammatory Bowel Disease: An Ecologic Analysis.” Inflammatory Bowel Diseases 17, no. 5: 1138–1145. 10.1002/ibd.21455. 20806342

[cph470022-bib-0007] Arcoleo, F. , S. Milano , P. D'Agostino , and E. Cillari . 1995. “Effect of Exogenous Leukotriene B4 (LTB4) on BALB/c Mice Splenocyte Production of Th1 and Th2 Lymphokines.” International Journal of Immunopharmacology 17, no. 6: 457–463. 10.1016/0192-0561(95)00038-4. 7499021

[cph470022-bib-0008] Arm, J. P. , S. P. O'Hickey , R. J. Hawksworth , et al. 1990. “Asthmatic Airways Have a Disproportionate Hyperresponsiveness to LTE4, as Compared With Normal Airways, but Not to LTC4, LTD4, Methacholine, and Histamine.” American Review of Respiratory Disease 142, no. 5: 1112–1118. 2173457 10.1164/ajrccm/142.5.1112

[cph470022-bib-0009] Bankova, L. G. , D. F. Dwyer , E. Yoshimoto , et al. 2018. “The Cysteinyl Leukotriene 3 Receptor Regulates Expansion of IL‐25‐Producing Airway Brush Cells Leading to Type 2 Inflammation.” Science Immunology 3, no. 28: eaat9453. 10.1126/sciimmunol.aat9453. 30291131 PMC6599626

[cph470022-bib-0010] Bankova, L. G. , J. Lai , E. Yoshimoto , et al. 2016. “Leukotriene E4 Elicits Respiratory Epithelial Cell Mucin Release Through the G‐Protein‐Coupled Receptor, GPR99.” Proceedings of the National Academy of Sciences of the United States of America 113, no. 22: 6242–6247. 10.1073/pnas.1605957113. 27185938 PMC4896673

[cph470022-bib-0011] Barajas‐Espinosa, A. , N. C. Ni , D. Yan , S. Zarini , R. C. Murphy , and C. D. Funk . 2012. “The Cysteinyl Leukotriene 2 Receptor Mediates Retinal Edema and Pathological Neovascularization in a Murine Model of Oxygen‐Induced Retinopathy.” FASEB Journal 26, no. 3: 1100–1109. 10.1096/fj.11-195792. 22131271

[cph470022-bib-0012] Barajas‐Espinosa, A. , F. Ochoa‐Cortes , M. P. Moos , F. D. Ramirez , S. J. Vanner , and C. D. Funk . 2011. “Characterization of the Cysteinyl Leukotriene 2 Receptor in Novel Expression Sites of the Gastrointestinal Tract.” American Journal of Pathology 178, no. 6: 2682–2689. 21641390 10.1016/j.ajpath.2011.02.041 PMC3124089

[cph470022-bib-0013] Bates, J. H. , M. Rincon , and C. G. Irvin . 2009. “Animal Models of Asthma.” American Journal of Physiology. Lung Cellular and Molecular Physiology 297, no. 3: L401–L410. 10.1152/ajplung.00027.2009. 19561139 PMC2739768

[cph470022-bib-0014] Baydi, Z. , Y. Limami , L. Khalki , et al. 2021. “An Update of Research Animal Models of Inflammatory Bowel Disease.” The Scientific World Journal 2021: 7479540. 10.1155/2021/7479540. 34938152 PMC8687830

[cph470022-bib-0015] Bernstein, C. N. , A. Wajda , and J. F. Blanchard . 2005. “The Clustering of Other Chronic Inflammatory Diseases in Inflammatory Bowel Disease: A Population‐Based Study.” Gastroenterology 129, no. 3: 827–836. 10.1053/j.gastro.2005.06.021. 16143122

[cph470022-bib-0016] Bhatt, L. , K. Roinestad , T. Van , and E. B. Springman . 2017. “Recent Advances in Clinical Development of Leukotriene B4 Pathway Drugs.” Seminars in Immunology 33: 65–73. 10.1016/j.smim.2017.08.007. 29042030

[cph470022-bib-0017] Biagioli, M. , S. Marchiano , C. di Giorgio , et al. 2023. “Combinatorial Targeting of G‐Protein‐Coupled Bile Acid Receptor 1 and Cysteinyl Leukotriene Receptor 1 Reveals a Mechanistic Role for Bile Acids and Leukotrienes in Drug‐Induced Liver Injury.” Hepatology 78, no. 1: 26–44. 10.1002/hep.32787. 36107019

[cph470022-bib-0018] Black, H. , M. Mendoza , and S. Murin . 2007. “Thoracic Manifestations of Inflammatory Bowel Disease.” Chest 131, no. 2: 524–532. 10.1378/chest.06-1074. 17296657

[cph470022-bib-0019] Blokzijl, H. , A. van Steenpaal , S. Vander Borght , et al. 2008. “Up‐Regulation and Cytoprotective Role of Epithelial Multidrug Resistance‐Associated Protein 1 in Inflammatory Bowel Disease.” Journal of Biological Chemistry 283, no. 51: 35630–35637. 10.1074/jbc.M804374200. 18838379

[cph470022-bib-0020] Brassard, P. , M. Vutcovici , P. Ernst , et al. 2015. “Increased Incidence of Inflammatory Bowel Disease in Quebec Residents With Airway Diseases.” European Respiratory Journal 45, no. 4: 962–968. 10.1183/09031936.00079414. 25406447

[cph470022-bib-0021] Budden, K. F. , S. L. Gellatly , D. L. Wood , et al. 2017. “Emerging Pathogenic Links Between Microbiota and the Gut‐Lung Axis.” Nature Reviews. Microbiology 15, no. 1: 55–63. 10.1038/nrmicro.2016.142. 27694885

[cph470022-bib-0022] Candelli, M. , L. Franza , G. Pignataro , et al. 2021. “Interaction Between Lipopolysaccharide and Gut Microbiota in Inflammatory Bowel Diseases.” International Journal of Molecular Sciences 22, no. 12: 6242. 10.3390/ijms22126242. 34200555 PMC8226948

[cph470022-bib-0023] Carter, B. Z. , Z. Z. Shi , R. Barrios , and M. W. Lieberman . 1998. “Gamma‐Glutamyl Leukotrienase, a Gamma‐Glutamyl Transpeptidase Gene Family Member, Is Expressed Primarily in Spleen.” Journal of Biological Chemistry 273, no. 43: 28277–28285. 9774450 10.1074/jbc.273.43.28277

[cph470022-bib-0024] Ceyhan, B. B. , S. Karakurt , H. Cevik , and M. Sungur . 2003. “Bronchial Hyperreactivity and Allergic Status in Inflammatory Bowel Disease.” Respiration 70, no. 1: 60–66. 10.1159/000068407. 12584393

[cph470022-bib-0025] Chao, K. L. , L. Kulakova , and O. Herzberg . 2017. “Gene Polymorphism Linked to Increased Asthma and IBD Risk Alters Gasdermin‐B Structure, a Sulfatide and Phosphoinositide Binding Protein.” Proceedings of the National Academy of Sciences of the United States of America 114, no. 7: E1128–E1137. 10.1073/pnas.1616783114. 28154144 PMC5321033

[cph470022-bib-0026] Chaudhuri, R. , V. Norris , K. Kelly , et al. 2014. “Effects of a FLAP Inhibitor, GSK2190915, in Asthmatics With High Sputum Neutrophils.” Pulmonary Pharmacology & Therapeutics 27, no. 1: 62–69. 10.1016/j.pupt.2013.11.007. 24333186

[cph470022-bib-0027] Chen, H. , X. Zhou , T. Liu , et al. 2025. “Postprandial Parasympathetic Signals Promote Lung Type 2 Immunity.” Neuron 113, no. 5: 670–683.e7. 10.1016/j.neuron.2024.12.020. 39837323

[cph470022-bib-0028] Chen, K. M. , K. P. Lan , and S. C. Lai . 2024. “Neuroprotective Effects of CysLT2R Antagonist on *Angiostrongylus cantonensis*‐Induced Edema and Meningoencephalitis.” Molecular and Biochemical Parasitology 260: 111649. 10.1016/j.molbiopara.2024.111649. 39004229

[cph470022-bib-0029] Cheng, Z. , Y. Zhou , X. Xiong , et al. 2024. “Traditional Herbal Pair Portulacae Herba and Granati Pericarpium Alleviates DSS‐Induced Colitis in Mice Through IL‐6/STAT3/SOCS3 Pathway.” Phytomedicine 126: 155283. 10.1016/j.phymed.2023.155283. 38422652

[cph470022-bib-0030] Christie, P. E. , R. Hawksworth , B. W. Spur , and T. H. Lee . 1992. “Effect of Indomethacin on leukotriene4‐Induced Histamine Hyperresponsiveness in Asthmatic Subjects.” American Review of Respiratory Disease 146, no. 6: 1506–1510. 1333740 10.1164/ajrccm/146.6.1506

[cph470022-bib-0031] Ciana, P. , M. Fumagalli , M. L. Trincavelli , et al. 2006. “The Orphan Receptor GPR17 Identified as a New Dual Uracil Nucleotides/Cysteinyl‐Leukotrienes Receptor.” EMBO Journal 25, no. 19: 4615–4627. 16990797 10.1038/sj.emboj.7601341 PMC1589991

[cph470022-bib-0032] Clark, J. D. , L. L. Lin , R. W. Kriz , et al. 1991. “A Novel Arachidonic Acid‐Selective Cytosolic PLA2 Contains a Ca(2+)‐Dependent Translocation Domain With Homology to PKC and GAP.” Cell 65, no. 6: 1043–1051. 1904318 10.1016/0092-8674(91)90556-e

[cph470022-bib-0033] Clarke, A. W. , L. Poulton , D. Shim , et al. 2018. “An Anti‐TL1A Antibody for the Treatment of Asthma and Inflammatory Bowel Disease.” MAbs 10, no. 4: 664–677. 10.1080/19420862.2018.1440164. 29436901 PMC5973687

[cph470022-bib-0034] Colazzo, F. , P. Gelosa , E. Tremoli , L. Sironi , and L. Castiglioni . 2017. “Role of the Cysteinyl Leukotrienes in the Pathogenesis and Progression of Cardiovascular Diseases.” Mediators of Inflammation 2017: 2432958. 10.1155/2017/2432958. 28932020 PMC5592403

[cph470022-bib-0035] Cole, A. T. , B. J. Pilkington , J. McLaughlan , C. Smith , M. Balsitis , and C. J. Hawkey . 1996. “Mucosal Factors Inducing Neutrophil Movement in Ulcerative Colitis: The Role of Interleukin 8 and Leukotriene B4.” Gut 39, no. 2: 248–254. 10.1136/gut.39.2.248. 8977339 PMC1383307

[cph470022-bib-0036] Davidson, A. B. , T. H. Lee , P. D. Scanlon , et al. 1987. “Bronchoconstrictor Effects of Leukotriene E4 in Normal and Asthmatic Subjects.” American Review of Respiratory Disease 135, no. 2: 333–337. 3028218 10.1164/arrd.1987.135.2.333

[cph470022-bib-0037] Derkacz, A. , P. Olczyk , K. Olczyk , and K. Komosinska‐Vassev . 2021. “The Role of Extracellular Matrix Components in Inflammatory Bowel Diseases.” Journal of Clinical Medicine 10, no. 5: 1122. 10.3390/jcm10051122. 33800267 PMC7962650

[cph470022-bib-0038] Devchand, P. R. , H. Keller , J. M. Peters , M. Vazquez , F. J. Gonzalez , and W. Wahli . 1996. “The PPARalpha‐Leukotriene B4 Pathway to Inflammation Control.” Nature 384, no. 6604: 39–43. 10.1038/384039a0. 8900274

[cph470022-bib-0039] Di Sabatino, A. , P. Biancheri , L. Rovedatti , T. T. Macdonald , and G. R. Corazza . 2012. “Recent Advances in Understanding Ulcerative Colitis.” Internal and Emergency Medicine 7, no. 2: 103–111. 10.1007/s11739-011-0719-z. 22068230

[cph470022-bib-0040] Dickson, R. P. , B. H. Singer , M. W. Newstead , et al. 2016. “Enrichment of the Lung Microbiome With Gut Bacteria in Sepsis and the Acute Respiratory Distress Syndrome.” Nature Microbiology 1, no. 10: 16113. 10.1038/nmicrobiol.2016.113. PMC5076472 27670109

[cph470022-bib-0041] Diehl, S. , and M. Rincon . 2002. “The Two Faces of IL‐6 on Th1/Th2 Differentiation.” Molecular Immunology 39, no. 9: 531–536. 10.1016/s0161-5890(02)00210-9. 12431386

[cph470022-bib-0042] Dixon, R. A. , R. E. Diehl , E. Opas , et al. 1990. “Requirement of a 5‐Lipoxygenase‐Activating Protein for Leukotriene Synthesis.” Nature 343, no. 6255: 282–284. 2300173 10.1038/343282a0

[cph470022-bib-0043] Doran, E. , F. Cai , C. T. J. Holweg , K. Wong , J. Brumm , and J. R. Arron . 2017. “Interleukin‐13 in Asthma and Other Eosinophilic Disorders.” Frontiers in Medicine 4: 139. 10.3389/fmed.2017.00139. 29034234 PMC5627038

[cph470022-bib-0044] Dore, E. , C. Joly‐Beauparlant , S. Morozumi , et al. 2022. “The Interaction of Secreted Phospholipase A2‐IIA With the Microbiota Alters Its Lipidome and Promotes Inflammation.” JCI Insight 7, no. 2: e152638. 10.1172/jci.insight.152638. 35076027 PMC8855825

[cph470022-bib-0045] Douglas, J. G. , C. F. McDonald , M. J. Leslie , J. Gillon , G. K. Crompton , and G. J. McHardy . 1989. “Respiratory Impairment in Inflammatory Bowel Disease: Does It Vary With Disease Activity?” Respiratory Medicine 83, no. 5: 389–394. 10.1016/s0954-6111(89)80070-8. 2616823

[cph470022-bib-0046] Drazen, J. M. , and K. F. Austen . 1987. “Leukotrienes and Airway Responses.” American Review of Respiratory Disease 136, no. 4: 985–998. 2821857 10.1164/ajrccm/136.4.985

[cph470022-bib-0047] Drazen, J. M. , K. F. Austen , R. A. Lewis , et al. 1980. “Comparative Airway and Vascular Activities of Leukotrienes C‐1 and D In Vivo and In Vitro.” Proceedings of the National Academy of Sciences of the United States of America 77, no. 7: 4354–4358. 10.1073/pnas.77.7.4354. 6933488 PMC349833

[cph470022-bib-0048] Drazen, J. M. , J. O'Brien , D. Sparrow , et al. 1992. “Recovery of Leukotriene E4 From the Urine of Patients With Airway Obstruction.” American Review of Respiratory Disease 146, no. 1: 104–108. 1320817 10.1164/ajrccm/146.1.104

[cph470022-bib-0049] Duah, E. , R. K. Adapala , N. Al‐Azzam , et al. 2013. “Cysteinyl Leukotrienes Regulate Endothelial Cell Inflammatory and Proliferative Signals Through CysLT(2) and CysLT(1) Receptors.” Scientific Reports 3: 3274. 10.1038/srep03274. 24253666 PMC3834363

[cph470022-bib-0050] Duah, E. , L. R. Teegala , V. Kondeti , et al. 2019. “Cysteinyl Leukotriene 2 Receptor Promotes Endothelial Permeability, Tumor Angiogenesis, and Metastasis.” Proceedings of the National Academy of Sciences of the United States of America 116, no. 1: 199–204. 10.1073/pnas.1817325115. 30559191 PMC6320507

[cph470022-bib-0051] Eade, O. E. , C. L. Smith , J. R. Alexander , and P. J. Whorwell . 1980. “Pulmonary Function in Patients With Inflammatory Bowel Disease.” American Journal of Gastroenterology 73, no. 2: 154–156. 6104919

[cph470022-bib-0052] Ekbom, A. , L. Brandt , F. Granath , C. G. Lofdahl , and A. Egesten . 2008. “Increased Risk of Both Ulcerative Colitis and Crohn's Disease in a Population Suffering From COPD.” Lung 186, no. 3: 167–172. 10.1007/s00408-008-9080-z. 18330638

[cph470022-bib-0053] Fang, L. , B. Adkins , V. Deyev , and E. R. Podack . 2008. “Essential Role of TNF Receptor Superfamily 25 (TNFRSF25) in the Development of Allergic Lung Inflammation.” Journal of Experimental Medicine 205, no. 5: 1037–1048. 10.1084/jem.20072528. 18411341 PMC2373837

[cph470022-bib-0054] Farrar, W. L. , and J. L. Humes . 1985. “The Role of Arachidonic Acid Metabolism in the Activities of Interleukin 1 and 2.” Journal of Immunology 135, no. 2: 1153–1159. 3924998

[cph470022-bib-0055] Figueroa, D. J. , L. Borish , D. Baramki , G. Philip , C. P. Austin , and J. F. Evans . 2003. “Expression of Cysteinyl Leukotriene Synthetic and Signalling Proteins in Inflammatory Cells in Active Seasonal Allergic Rhinitis.” Clinical and Experimental Allergy 33, no. 10: 1380–1388. 14519144 10.1046/j.1365-2222.2003.01786.x

[cph470022-bib-0056] Fornasa, G. , K. Tsilingiri , F. Caprioli , et al. 2015. “Dichotomy of Short and Long Thymic Stromal Lymphopoietin Isoforms in Inflammatory Disorders of the Bowel and Skin.” Journal of Allergy and Clinical Immunology 136, no. 2: 413–422. 10.1016/j.jaci.2015.04.011. 26014813 PMC4534776

[cph470022-bib-0057] Furusawa, Y. , Y. Obata , S. Fukuda , et al. 2013. “Commensal Microbe‐Derived Butyrate Induces the Differentiation of Colonic Regulatory T Cells.” Nature 504, no. 7480: 446–450. 10.1038/nature12721. 24226770

[cph470022-bib-0058] Fuss, I. J. , F. Heller , M. Boirivant , et al. 2004. “Nonclassical CD1d‐Restricted NK T Cells That Produce IL‐13 Characterize an Atypical Th2 Response in Ulcerative Colitis.” Journal of Clinical Investigation 113, no. 10: 1490–1497. 10.1172/JCI19836. 15146247 PMC406524

[cph470022-bib-0059] Gans, M. D. , and T. Gavrilova . 2020. “Understanding the Immunology of Asthma: Pathophysiology, Biomarkers, and Treatments for Asthma Endotypes.” Paediatric Respiratory Reviews 36: 118–127. 10.1016/j.prrv.2019.08.002. 31678040

[cph470022-bib-0060] Garg, P. , M. Vijay‐Kumar , L. Wang , A. T. Gewirtz , D. Merlin , and S. V. Sitaraman . 2009. “Matrix Metalloproteinase‐9‐Mediated Tissue Injury Overrides the Protective Effect of Matrix Metalloproteinase‐2 During Colitis.” American Journal of Physiology. Gastrointestinal and Liver Physiology 296, no. 2: G175–G184. 10.1152/ajpgi.90454.2008. 19171847 PMC2643910

[cph470022-bib-0061] Gauvreau, G. M. , L. P. Boulet , J. M. FitzGerald , et al. 2016. “A Dual CysLT(1/2) Antagonist Attenuates Allergen‐Induced Airway Responses in Subjects With Mild Allergic Asthma.” Allergy 71, no. 12: 1721–1727. 10.1111/all.12987. 27444660

[cph470022-bib-0062] Gauvreau, G. M. , K. N. Parameswaran , R. M. Watson , and P. M. O'Byrne . 2001. “Inhaled Leukotriene E(4), but Not Leukotriene D(4), Increased Airway Inflammatory Cells in Subjects With Atopic Asthma.” American Journal of Respiratory and Critical Care Medicine 164, no. 8 Pt 1: 1495–1500. 11704602 10.1164/ajrccm.164.8.2102033

[cph470022-bib-0063] Gertner, D. J. , D. S. Rampton , M. V. Madden , I. C. Talbot , R. J. Nicholls , and J. E. Lennard‐Jones . 1994. “Increased Leukotriene B4 Release From Ileal Pouch Mucosa in Ulcerative Colitis Compared With Familial Adenomatous Polyposis.” Gut 35, no. 10: 1429–1432. 10.1136/gut.35.10.1429. 7959200 PMC1375019

[cph470022-bib-0064] Ghorbanzadeh, B. , M. A. Behmanesh , R. Mahmoudinejad , M. Zamaniyan , S. Ekhtiar , and Y. Paridar . 2022. “The Effect of Montelukast, a Leukotriene Receptor Antagonist, on the Acetic Acid‐Induced Model of Colitis in Rats: Involvement of NO‐cGMP‐K(ATP) Channels Pathway.” Frontiers in Pharmacology 13: 1011141. 10.3389/fphar.2022.1011141. 36225573 PMC9549743

[cph470022-bib-0065] Gray, J. , K. Oehrle , G. Worthen , T. Alenghat , J. Whitsett , and H. Deshmukh . 2017. “Intestinal Commensal Bacteria Mediate Lung Mucosal Immunity and Promote Resistance of Newborn Mice to Infection.” Science Translational Medicine 9, no. 376: eaaf9412. 10.1126/scitranslmed.aaf9412. 28179507 PMC5880204

[cph470022-bib-0066] Gudneppanavar, R. , S. E. E. Kattuman , L. R. Teegala , et al. 2023. “Epigenetic Histone Modification by Butyrate Downregulates KIT and Attenuates Mast Cell Function.” Journal of Cellular and Molecular Medicine 27, no. 19: 2983–2994. 10.1111/jcmm.17924. 37603611 PMC10538265

[cph470022-bib-0067] Hadjigol, S. , K. G. Netto , S. Maltby , et al. 2020. “Lipopolysaccharide Induces Steroid‐Resistant Exacerbations in a Mouse Model of Allergic Airway Disease Collectively Through IL‐13 and Pulmonary Macrophage Activation.” Clinical and Experimental Allergy 50, no. 1: 82–94. 10.1111/cea.13505. 31579973

[cph470022-bib-0068] Haldar, S. , S. R. Jadhav , V. Gulati , et al. 2023. “Unravelling the Gut‐Lung Axis: Insights Into Microbiome Interactions and Traditional Indian Medicine's Perspective on Optimal Health.” FEMS Microbiology Ecology 99, no. 10: fiad103. 10.1093/femsec/fiad103. 37656879 PMC10508358

[cph470022-bib-0069] Hallstrand, T. S. , and W. R. Henderson Jr. 2010. “An Update on the Role of Leukotrienes in Asthma.” Current Opinion in Allergy and Clinical Immunology 10, no. 1: 60–66. 10.1097/ACI.0b013e32833489c3. 19915456 PMC2838730

[cph470022-bib-0070] Halonen, J. I. , T. Lanki , T. Yli‐Tuomi , M. Kulmala , P. Tiittanen , and J. Pekkanen . 2008. “Urban Air Pollution, and Asthma and COPD Hospital Emergency Room Visits.” Thorax 63, no. 7: 635–641. 10.1136/thx.2007.091371. 18267984

[cph470022-bib-0071] Hammerbeck, D. M. , and D. R. Brown . 1996. “Presence of Immunocytes and Sulfidopeptide Leukotrienes in the Inflamed Guinea Pig Distal Colon.” Inflammation 20, no. 4: 413–425. 10.1007/BF01486743. 8872504

[cph470022-bib-0072] Hansson, G. C. 2019. “Mucus and Mucins in Diseases of the Intestinal and Respiratory Tracts.” Journal of Internal Medicine 285, no. 5: 479–490. 10.1111/joim.12910. 30963635 PMC6497544

[cph470022-bib-0073] He, R. , Y. Chen , and Q. Cai . 2020. “The Role of the LTB4‐BLT1 Axis in Health and Disease.” Pharmacological Research 158: 104857. 10.1016/j.phrs.2020.104857. 32439596

[cph470022-bib-0074] Heise, C. E. , B. F. O'Dowd , D. J. Figueroa , et al. 2000. “Characterization of the Human Cysteinyl Leukotriene 2 Receptor.” Journal of Biological Chemistry 275, no. 39: 30531–30536. 10851239 10.1074/jbc.M003490200

[cph470022-bib-0075] Hemminki, K. , X. Li , J. Sundquist , and K. Sundquist . 2010. “Subsequent Autoimmune or Related Disease in Asthma Patients: Clustering of Diseases or Medical Care?” Annals of Epidemiology 20, no. 3: 217–222. 10.1016/j.annepidem.2009.11.007. 20036578

[cph470022-bib-0076] Hennig, R. , X. Z. Ding , W. G. Tong , R. C. Witt , B. D. Jovanovic , and T. E. Adrian . 2004. “Effect of LY293111 in Combination With Gemcitabine in Colonic Cancer.” Cancer Letters 210, no. 1: 41–46. 10.1016/j.canlet.2004.02.023. 15172119

[cph470022-bib-0077] Hennig, R. , J. Ventura , R. Segersvard , et al. 2005. “LY293111 Improves Efficacy of Gemcitabine Therapy on Pancreatic Cancer in a Fluorescent Orthotopic Model in Athymic Mice.” Neoplasia 7, no. 4: 417–425. 10.1593/neo.04559. 15967119 PMC1501143

[cph470022-bib-0078] Hoffman, B. C. , and N. Rabinovitch . 2018. “Urinary Leukotriene E(4) as a Biomarker of Exposure, Susceptibility, and Risk in Asthma: An Update.” Immunology and Allergy Clinics of North America 38, no. 4: 599–610. 10.1016/j.iac.2018.06.011. 30342582

[cph470022-bib-0079] Iizuka, Y. , T. Okuno , K. Saeki , et al. 2010. “Protective Role of the Leukotriene B4 Receptor BLT2 in Murine Inflammatory Colitis.” FASEB Journal 24, no. 12: 4678–4690. 10.1096/fj.10-165050. 20667973

[cph470022-bib-0080] Jacobsen, H. A. , A. Karachalia Sandri , U. M. Weinreich , T. Jess , and L. Larsen . 2024. “Increased Risk of Obstructive Lung Disease in Inflammatory Bowel Disease: A Population‐Based Cohort Study.” United European Gastroenterology Journal 12, no. 4: 477–486. 10.1002/ueg2.12527. 38183388 PMC11091783

[cph470022-bib-0081] Jo‐Watanabe, A. , T. Okuno , and T. Yokomizo . 2019. “The Role of Leukotrienes as Potential Therapeutic Targets in Allergic Disorders.” International Journal of Molecular Sciences 20, no. 14: 3580. 10.3390/ijms20143580. 31336653 PMC6679143

[cph470022-bib-0082] Kabata, H. , A. L. Flamar , T. Mahlakoiv , et al. 2020. “Targeted Deletion of the TSLP Receptor Reveals Cellular Mechanisms That Promote Type 2 Airway Inflammation.” Mucosal Immunology 13, no. 4: 626–636. 10.1038/s41385-020-0266-x. 32066836 PMC7311324

[cph470022-bib-0083] Kanaoka, Y. , and J. A. Boyce . 2004. “Cysteinyl Leukotrienes and Their Receptors: Cellular Distribution and Function in Immune and Inflammatory Responses.” Journal of Immunology 173, no. 3: 1503–1510. 10.4049/jimmunol.173.3.1503 15265876

[cph470022-bib-0084] Kanaoka, Y. , A. Maekawa , and K. F. Austen . 2013. “Identification of GPR99 Protein as a Potential Third Cysteinyl Leukotriene Receptor With a Preference for Leukotriene E4 Ligand.” Journal of Biological Chemistry 288, no. 16: 10967–10972. 23504326 10.1074/jbc.C113.453704 PMC3630866

[cph470022-bib-0085] Kasarello, K. , A. Cudnoch‐Jedrzejewska , and K. Czarzasta . 2023. “Communication of Gut Microbiota and Brain via Immune and Neuroendocrine Signaling.” Frontiers in Microbiology 14: 1118529. 10.3389/fmicb.2023.1118529. 36760508 PMC9907780

[cph470022-bib-0086] Keely, S. , N. J. Talley , and P. M. Hansbro . 2012. “Pulmonary‐Intestinal Cross‐Talk in Mucosal Inflammatory Disease.” Mucosal Immunology 5, no. 1: 7–18. 10.1038/mi.2011.55. 22089028 PMC3243663

[cph470022-bib-0087] Kelly, E. A. , and N. N. Jarjour . 2003. “Role of Matrix Metalloproteinases in Asthma.” Current Opinion in Pulmonary Medicine 9, no. 1: 28–33. 10.1097/00063198-200301000-00005. 12476081

[cph470022-bib-0088] Kikut, J. , M. Mokrzycka , A. Drozd , U. Grzybowska‐Chlebowczyk , M. Zietek , and M. Szczuko . 2022. “Involvement of Proinflammatory Arachidonic Acid (ARA) Derivatives in Crohn's Disease (CD) and Ulcerative Colitis (UC).” Journal of Clinical Medicine 11, no. 7: 1861. 10.3390/jcm11071861. 35407469 PMC8999554

[cph470022-bib-0089] Kim, Y. G. , K. G. Udayanga , N. Totsuka , J. B. Weinberg , G. Nunez , and A. Shibuya . 2014. “Gut Dysbiosis Promotes M2 Macrophage Polarization and Allergic Airway Inflammation via Fungi‐Induced PGE(2).” Cell Host & Microbe 15, no. 1: 95–102. 10.1016/j.chom.2013.12.010. 24439901 PMC3957200

[cph470022-bib-0090] Kishino, S. , M. Takeuchi , S. B. Park , et al. 2013. “Polyunsaturated Fatty Acid Saturation by Gut Lactic Acid Bacteria Affecting Host Lipid Composition.” Proceedings of the National Academy of Sciences of the United States of America 110, no. 44: 17808–17813. 10.1073/pnas.1312937110. 24127592 PMC3816446

[cph470022-bib-0091] Ko, J. K. , and C. H. Cho . 2005. “The Diverse Actions of Nicotine and Different Extracted Fractions From Tobacco Smoke Against Hapten‐Induced Colitis in Rats.” Toxicological Sciences 87, no. 1: 285–295. 10.1093/toxsci/kfi238. 15976189

[cph470022-bib-0092] Kondeti, V. , N. Al‐Azzam , E. Duah , C. K. Thodeti , J. A. Boyce , and S. Paruchuri . 2016. “Leukotriene D4 and Prostaglandin E2 Signals Synergize and Potentiate Vascular Inflammation in a Mast Cell‐Dependent Manner Through Cysteinyl Leukotriene Receptor 1 and E‐Prostanoid Receptor 3.” Journal of Allergy and Clinical Immunology 137, no. 1: 289–298. 10.1016/j.jaci.2015.06.030. 26255103 PMC5839097

[cph470022-bib-0093] Kraft, S. C. , R. H. Earle , M. Roesler , and J. R. Esterly . 1976. “Unexplained Bronchopulmonary Disease With Inflammatory Bowel Disease.” Archives of Internal Medicine 136, no. 4: 454–459. 1267553

[cph470022-bib-0094] Kuenzig, M. E. , C. Barnabe , C. H. Seow , et al. 2017. “Asthma Is Associated With Subsequent Development of Inflammatory Bowel Disease: A Population‐Based Case‐Control Study.” Clinical Gastroenterology and Hepatology 15, no. 9: 1405–1412. e3. 10.1016/j.cgh.2017.02.042. 28344063

[cph470022-bib-0095] Labarca, G. , L. Drake , G. Horta , et al. 2019. “Association Between Inflammatory Bowel Disease and Chronic Obstructive Pulmonary Disease: A Systematic Review and Meta‐Analysis.” BMC Pulmonary Medicine 19, no. 1: 186. 10.1186/s12890-019-0963-y. 31660921 PMC6819559

[cph470022-bib-0096] Laitinen, L. A. , A. Laitinen , T. Haahtela , V. Vilkka , B. W. Spur , and T. H. Lee . 1993. “Leukotriene E4 and Granulocytic Infiltration Into Asthmatic Airways.” Lancet 341, no. 8851: 989–990. 8096945 10.1016/0140-6736(93)91073-u

[cph470022-bib-0097] Lam, B. K. , J. F. Penrose , G. J. Freeman , and K. F. Austen . 1994. “Expression Cloning of a cDNA for Human Leukotriene C4 Synthase, an Integral Membrane Protein Conjugating Reduced Glutathione to Leukotriene A4.” Proceedings of the National Academy of Sciences of the United States of America 91, no. 16: 7663–7667. 8052639 10.1073/pnas.91.16.7663 PMC44462

[cph470022-bib-0098] Le, U. , and A. W. Burks . 2014. “Antibiotic Exposure in the First Two Years of Life and Development of Asthma and Other Allergic Diseases by 7.5 Yr: A Dose‐Dependent Relationship.” Pediatrics 134, no. S3: S135. 10.1542/peds.2014-1817E. 25363911

[cph470022-bib-0099] Lee, A. J. , M. Ro , K. J. Cho , and J. H. Kim . 2017. “Lipopolysaccharide/TLR4 Stimulates IL‐13 Production Through a MyD88‐BLT2‐Linked Cascade in Mast Cells, Potentially Contributing to the Allergic Response.” Journal of Immunology 199, no. 2: 409–417. 10.4049/jimmunol.1602062. 28600286

[cph470022-bib-0100] Lee, C. W. , R. A. Lewis , E. J. Corey , and K. F. Austen . 1983. “Conversion of Leukotriene D4 to Leukotriene E4 by a Dipeptidase Released From the Specific Granule of Human Polymorphonuclear Leucocytes.” Immunology 48, no. 1: 27–35. 6293969 PMC1453997

[cph470022-bib-0101] Lee, T. H. , K. F. Austen , E. J. Corey , and J. M. Drazen . 1984. “Leukotriene E4‐Induced Airway Hyperresponsiveness of Guinea Pig Tracheal Smooth Muscle to Histamine and Evidence for Three Separate Sulfidopeptide Leukotriene Receptors.” Proceedings of the National Academy of Sciences of the United States of America 81, no. 15: 4922–4925. 6087352 10.1073/pnas.81.15.4922 PMC391604

[cph470022-bib-0102] Lees, C. W. , J. C. Barrett , M. Parkes , and J. Satsangi . 2011. “New IBD Genetics: Common Pathways With Other Diseases.” Gut 60, no. 12: 1739–1753. 10.1136/gut.2009.199679. 21300624

[cph470022-bib-0103] Leier, I. , G. Jedlitschky , U. Buchholz , S. P. Cole , R. G. Deeley , and D. Keppler . 1994. “The MRP Gene Encodes an ATP‐Dependent Export Pump for Leukotriene C4 and Structurally Related Conjugates.” Journal of Biological Chemistry 269, no. 45: 27807–27810. 7961706

[cph470022-bib-0104] Leslie, W. D. , N. Miller , L. Rogala , and C. N. Bernstein . 2008. “Vitamin D Status and Bone Density in Recently Diagnosed Inflammatory Bowel Disease: The Manitoba IBD Cohort Study.” American Journal of Gastroenterology 103, no. 6: 1451–1459. 10.1111/j.1572-0241.2007.01753.x. 18422819

[cph470022-bib-0105] Li, C. , W. Chen , F. Lin , et al. 2023. “Functional Two‐Way Crosstalk Between Brain and Lung: The Brain‐Lung Axis.” Cellular and Molecular Neurobiology 43, no. 3: 991–1003. 10.1007/s10571-022-01238-z. 35678887 PMC9178545

[cph470022-bib-0106] Liang, S. , Z. Zhou , Z. Zhou , et al. 2022. “CBX4 Regulates Long‐Form Thymic Stromal Lymphopoietin‐Mediated Airway Inflammation Through SUMOylation in House Dust Mite‐Induced Asthma.” American Journal of Respiratory Cell and Molecular Biology 66, no. 6: 648–660. 10.1165/rcmb.2021-0301OC. 35358396

[cph470022-bib-0107] Lin, K. , S. Fang , B. Cai , et al. 2014. “ERK/Egr‐1 Signaling Pathway Is Involved in CysLT2 Receptor‐Mediated IL‐8 Production in HEK293 Cells.” European Journal of Cell Biology 93, no. 7: 278–288. 10.1016/j.ejcb.2014.05.001. 24925646

[cph470022-bib-0108] Liu, M. , K. Saeki , T. Matsunobu , et al. 2014. “12‐Hydroxyheptadecatrienoic Acid Promotes Epidermal Wound Healing by Accelerating Keratinocyte Migration via the BLT2 Receptor.” Journal of Experimental Medicine 211, no. 6: 1063–1078. 10.1084/jem.20132063. 24821912 PMC4042643

[cph470022-bib-0109] Liu, M. C. , L. M. Dube , and J. Lancaster . 1996. “Acute and Chronic Effects of a 5‐Lipoxygenase Inhibitor in Asthma: A 6‐Month Randomized Multicenter Trial. Zileuton Study Group.” Journal of Allergy and Clinical Immunology 98, no. 5 Pt 1: 859–871. 10.1016/s0091-6749(96)80002-9. 8939149

[cph470022-bib-0110] Louis, E. , R. Louis , V. Drion , et al. 1995. “Increased Frequency of Bronchial Hyperresponsiveness in Patients With Inflammatory Bowel Disease.” Allergy 50, no. 9: 729–733. 10.1111/j.1398-9995.1995.tb01214.x. 8546267

[cph470022-bib-0111] Luthers, C. R. , T. M. Dunn , and A. L. Snow . 2020. “ORMDL3 and Asthma: Linking Sphingolipid Regulation to Altered T Cell Function.” Frontiers in Immunology 11: 597945. 10.3389/fimmu.2020.597945. 33424845 PMC7793773

[cph470022-bib-0112] Lynch, K. R. , G. P. O'Neill , Q. Liu , et al. 1999. “Characterization of the Human Cysteinyl Leukotriene CysLT1 Receptor.” Nature 399, no. 6738: 789–793. 10391245 10.1038/21658

[cph470022-bib-0113] Madan, J. C. , D. C. Koestler , B. A. Stanton , et al. 2012. “Serial Analysis of the Gut and Respiratory Microbiome in Cystic Fibrosis in Infancy: Interaction Between Intestinal and Respiratory Tracts and Impact of Nutritional Exposures.” MBio 3, no. 4: e00251‐12. 10.1128/mBio.00251-12. PMC3428694 22911969

[cph470022-bib-0114] Maekawa, A. , B. Balestrieri , K. F. Austen , and Y. Kanaoka . 2009. “GPR17 Is a Negative Regulator of the Cysteinyl Leukotriene 1 Receptor Response to Leukotriene D4.” Proceedings of the National Academy of Sciences of the United States of America 106, no. 28: 11685–11690. 19561298 10.1073/pnas.0905364106 PMC2710631

[cph470022-bib-0115] Mahgoub, A. A. , A. A. El‐Medany , H. H. Hager , A. A. Mustafa , and D. M. El‐Sabah . 2003. “Evaluating the Prophylactic Potential of Zafirlukast Against the Toxic Effects of Acetic Acid on the Rat Colon.” Toxicology Letters 145, no. 1: 79–87. 10.1016/s0378-4274(03)00269-8. 12962976

[cph470022-bib-0116] Malaviya, R. , R. Malaviya , and B. A. Jakschik . 1993. “Reversible Translocation of 5‐Lipoxygenase in Mast Cells Upon IgE/Antigen Stimulation.” Journal of Biological Chemistry 268, no. 7: 4939–4944. 8444871

[cph470022-bib-0117] Marini, M. , E. Vittori , J. Hollemborg , and S. Mattoli . 1992. “Expression of the Potent Inflammatory Cytokines, Granulocyte‐Macrophage‐Colony‐Stimulating Factor and Interleukin‐6 and Interleukin‐8, in Bronchial Epithelial Cells of Patients With Asthma.” Journal of Allergy and Clinical Immunology 89, no. 5: 1001–1009. 10.1016/0091-6749(92)90223-o. 1583242

[cph470022-bib-0118] Marvisi, M. , P. D. Borrello , M. Brianti , G. Fornarsari , G. Marani , and A. Guariglia . 2000. “Changes in the Carbon Monoxide Diffusing Capacity of the Lung in Ulcerative Colitis.” European Respiratory Journal 16, no. 5: 965–968. 10.1183/09031936.00.16596500. 11153600

[cph470022-bib-0119] Merten, N. , J. Fischer , K. Simon , et al. 2018. “Repurposing HAMI3379 to Block GPR17 and Promote Rodent and Human Oligodendrocyte Differentiation.” Cell Chemical Biology 25, no. 6: 775–786. e5. 10.1016/j.chembiol.2018.03.012. 29706593 PMC6685917

[cph470022-bib-0120] Meylan, F. , E. T. Hawley , L. Barron , et al. 2014. “The TNF‐Family Cytokine TL1A Promotes Allergic Immunopathology Through Group 2 Innate Lymphoid Cells.” Mucosal Immunology 7, no. 4: 958–968. 10.1038/mi.2013.114. 24368564 PMC4165592

[cph470022-bib-0121] Meylan, F. , Y. J. Song , I. Fuss , et al. 2011. “The TNF‐Family Cytokine TL1A Drives IL‐13‐Dependent Small Intestinal Inflammation.” Mucosal Immunology 4, no. 2: 172–185. 10.1038/mi.2010.67. 20980995 PMC3437258

[cph470022-bib-0122] Miehsler, W. , W. Reinisch , E. Valic , et al. 2004. “Is Inflammatory Bowel Disease an Independent and Disease Specific Risk Factor for Thromboembolism?” Gut 53, no. 4: 542–548. 10.1136/gut.2003.025411. 15016749 PMC1773996

[cph470022-bib-0123] Miyahara, N. , K. Takeda , S. Miyahara , et al. 2005. “Requirement for Leukotriene B4 Receptor 1 in Allergen‐Induced Airway Hyperresponsiveness.” American Journal of Respiratory and Critical Care Medicine 172, no. 2: 161–167. 10.1164/rccm.200502-205OC. 15849325 PMC2718465

[cph470022-bib-0124] Modi, S. R. , J. J. Collins , and D. A. Relman . 2014. “Antibiotics and the Gut Microbiota.” Journal of Clinical Investigation 124, no. 10: 4212–4218. 10.1172/JCI72333. 25271726 PMC4191029

[cph470022-bib-0125] Motley, R. J. , J. Rhodes , G. Williams , I. A. Tavares , and A. Bennett . 1990. “Smoking, Eicosanoids and Ulcerative Colitis.” Journal of Pharmacy and Pharmacology 42, no. 4: 288–289. 10.1111/j.2042-7158.1990.tb05411.x. 1974301

[cph470022-bib-0126] Narasimhan, K. 2021. “Difficult to Treat and Severe Asthma: Management Strategies.” American Family Physician 103, no. 5: 286–290. 33630543

[cph470022-bib-0127] Neveu, W. A. , E. Bernardo , J. L. Allard , et al. 2011. “Fungal Allergen β‐Glucans Trigger p38 Mitogen‐Activated Protein Kinase–Mediated IL‐6 Translation in Lung Epithelial Cells.” American Journal of Respiratory Cell and Molecular Biology 45, no. 6: 1133–1141. 10.1165/rcmb.2011-0054OC. 21642586 PMC3262672

[cph470022-bib-0128] Ni, N. C. , D. Yan , L. L. Ballantyne , et al. 2011. “A Selective Cysteinyl Leukotriene Receptor 2 Antagonist Blocks Myocardial Ischemia/Reperfusion Injury and Vascular Permeability in Mice.” Journal of Pharmacology and Experimental Therapeutics 339, no. 3: 768–778. 10.1124/jpet.111.186031. 21903747

[cph470022-bib-0129] Nicholson, D. W. , A. Ali , J. P. Vaillancourt , et al. 1993. “Purification to Homogeneity and the N‐Terminal Sequence of Human Leukotriene C4 Synthase: A Homodimeric Glutathione S‐Transferase Composed of 18‐kDa Subunits.” Proceedings of the National Academy of Sciences of the United States of America 90, no. 5: 2015–2019. 8446623 10.1073/pnas.90.5.2015 PMC46011

[cph470022-bib-0130] Nicosia, S. , V. Capra , and G. E. Rovati . 2001. “Leukotrienes as Mediators of Asthma.” Pulmonary Pharmacology & Therapeutics 14, no. 1: 3–19. 10.1006/pupt.2000.0262. 11162414

[cph470022-bib-0131] Nowarski, R. , R. Jackson , N. Gagliani , et al. 2015. “Epithelial IL‐18 Equilibrium Controls Barrier Function in Colitis.” Cell 163, no. 6: 1444–1456. 10.1016/j.cell.2015.10.072. 26638073 PMC4943028

[cph470022-bib-0132] O'Driscoll, B. R. , O. Cromwell , and A. B. Kay . 1984. “Sputum Leukotrienes in Obstructive Airways Diseases.” Clinical and Experimental Immunology 55, no. 2: 397–404. 6321071 PMC1535807

[cph470022-bib-0133] Ohata, A. , M. Usami , and M. Miyoshi . 2005. “Short‐Chain Fatty Acids Alter Tight Junction Permeability in Intestinal Monolayer Cells via Lipoxygenase Activation.” Nutrition 21, no. 7–8: 838–847. 10.1016/j.nut.2004.12.004. 15975492

[cph470022-bib-0134] O'Hickey, S. P. , R. J. Hawksworth , C. Y. Fong , J. P. Arm , B. W. Spur , and T. H. Lee . 1991. “Leukotrienes C4, D4, and E4 Enhance Histamine Responsiveness in Asthmatic Airways.” American Review of Respiratory Disease 144, no. 5: 1053–1057. 1659268 10.1164/ajrccm/144.5.1053

[cph470022-bib-0135] Okuno, T. , Y. Iizuka , H. Okazaki , T. Yokomizo , R. Taguchi , and T. Shimizu . 2008. “12(S)‐Hydroxyheptadeca‐5Z, 8E, 10E‐Trienoic Acid Is a Natural Ligand for Leukotriene B4 Receptor 2.” Journal of Experimental Medicine 205, no. 4: 759–766. 10.1084/jem.20072329. 18378794 PMC2292216

[cph470022-bib-0136] Ordas, I. , L. Eckmann , M. Talamini , D. C. Baumgart , and W. J. Sandborn . 2012. “Ulcerative Colitis.” Lancet 380, no. 9853: 1606–1619. 10.1016/S0140-6736(12)60150-0. 22914296

[cph470022-bib-0137] Panettieri, R. A., Jr. 1998. “Cellular and Molecular Mechanisms Regulating Airway Smooth Muscle Proliferation and Cell Adhesion Molecule Expression.” American Journal of Respiratory and Critical Care Medicine 158, no. 5 pt. 3: S133–S140. 10.1164/ajrccm.158.supplement_2.13tac900. 9817736

[cph470022-bib-0138] Papanikolaou, I. , K. Kagouridis , and S. A. Papiris . 2014. “Patterns of Airway Involvement in Inflammatory Bowel Diseases.” World Journal of Gastrointestinal Pathophysiology 5, no. 4: 560–569. 10.4291/wjgp.v5.i4.560. 25400999 PMC4231520

[cph470022-bib-0139] Park, J. H. , D. Y. Jeong , L. Peyrin‐Biroulet , M. Eisenhut , and J. I. Shin . 2017. “Insight Into the Role of TSLP in Inflammatory Bowel Diseases.” Autoimmunity Reviews 16, no. 1: 55–63. 10.1016/j.autrev.2016.09.014. 27697608

[cph470022-bib-0140] Paruchuri, S. , Y. Jiang , C. Feng , S. A. Francis , J. Plutzky , and J. A. Boyce . 2008. “Leukotriene E4 Activates Peroxisome Proliferator‐Activated Receptor Gamma and Induces Prostaglandin D2 Generation by Human Mast Cells.” Journal of Biological Chemistry 283, no. 24: 16477–16487. 18411276 10.1074/jbc.M705822200 PMC2423236

[cph470022-bib-0141] Paruchuri, S. , H. Tashimo , C. Feng , et al. 2009. “Leukotriene E4‐Induced Pulmonary Inflammation Is Mediated by the P2Y12 Receptor.” Journal of Experimental Medicine 206, no. 11: 2543–2555. 19822647 10.1084/jem.20091240 PMC2768854

[cph470022-bib-0142] Pasquis, P. , R. Colin , P. Denis , P. Baptiste , J. P. Galmiche , and P. Hecketsweiler . 1981. “Transient Pulmonary Impairment During Attacks of Crohn's Disease.” Respiration 41, no. 1: 56–59. 10.1159/000194359. 7244392

[cph470022-bib-0143] Pastorelli, L. , R. R. Garg , S. B. Hoang , et al. 2010. “Epithelial‐Derived IL‐33 and Its Receptor ST2 Are Dysregulated in Ulcerative Colitis and in Experimental Th1/Th2 Driven Enteritis.” Proceedings of the National Academy of Sciences of the United States of America 107, no. 17: 8017–8022. 10.1073/pnas.0912678107. 20385815 PMC2867895

[cph470022-bib-0144] Persson, P. G. , O. Bernell , C. E. Leijonmarck , B. Y. Farahmand , G. Hellers , and A. Ahlbom . 1996. “Survival and Cause‐Specific Mortality in Inflammatory Bowel Disease: A Population‐Based Cohort Study.” Gastroenterology 110, no. 5: 1339–1345. 10.1053/gast.1996.v110.pm8613037. 8613037

[cph470022-bib-0145] Peskar, B. M. , K. W. Dreyling , B. A. Peskar , B. May , and H. Goebell . 1986. “Enhanced Formation of Sulfidopeptide‐Leukotrienes in Ulcerative Colitis and Crohn's Disease: Inhibition by Sulfasalazine and 5‐Aminosalicylic Acid.” Agents and Actions 18, no. 3–4: 381–383. 10.1007/BF01965001. 2875632

[cph470022-bib-0146] Pu, Q. , P. Lin , P. Gao , et al. 2021. “Gut Microbiota Regulate Gut‐Lung Axis Inflammatory Responses by Mediating ILC2 Compartmental Migration.” Journal of Immunology 207, no. 1: 257–267. 10.4049/jimmunol.2001304. PMC8674377 34135060

[cph470022-bib-0147] Qian, G. , W. Jiang , B. Zou , et al. 2018. “LPS Inactivation by a Host Lipase Allows Lung Epithelial Cell Sensitization for Allergic Asthma.” Journal of Experimental Medicine 215, no. 9: 2397–2412. 10.1084/jem.20172225. 30021797 PMC6122967

[cph470022-bib-0148] Reed, C. E. , and H. Kita . 2004. “The Role of Protease Activation of Inflammation in Allergic Respiratory Diseases.” Journal of Allergy and Clinical Immunology 114, no. 5: 997–1008. 10.1016/j.jaci.2004.07.060. 15536399

[cph470022-bib-0149] Risnes, K. R. , K. Belanger , W. Murk , and M. B. Bracken . 2011. “Antibiotic Exposure by 6 Months and Asthma and Allergy at 6 Years: Findings in a Cohort of 1,401 US Children.” American Journal of Epidemiology 173, no. 3: 310–318. 10.1093/aje/kwq400. 21190986 PMC3105273

[cph470022-bib-0150] Rohn, T. A. , S. Numao , H. Otto , C. Loesche , and G. Thoma . 2021. “Drug Discovery Strategies for Novel Leukotriene A4 Hydrolase Inhibitors.” Expert Opinion on Drug Discovery 16, no. 12: 1483–1495. 10.1080/17460441.2021.1948998. 34191664

[cph470022-bib-0151] Rola‐Pleszczynski, M. , P. A. Chavaillaz , and I. Lemaire . 1986. “Stimulation of Interleukin 2 and Interferon Gamma Production by Leukotriene B4 in Human Lymphocyte Cultures.” Prostaglandins, Leukotrienes, and Medicine 23, no. 2–3: 207–210. 10.1016/0262-1746(86)90187-3. 3020587

[cph470022-bib-0152] Rustgi, S. D. , M. Kayal , and S. C. Shah . 2020. “Sex‐Based Differences in Inflammatory Bowel Diseases: A Review.” Therapeutic Advances in Gastroenterology 13: 1756284820915043. 10.1177/1756284820915043. 32523620 PMC7236567

[cph470022-bib-0153] Schumert, R. , J. Towner , and R. D. Zipser . 1988. “Role of Eicosanoids in Human and Experimental Colitis.” Digestive Diseases and Sciences 33, no. 3 Suppl: 58S–64S. 10.1007/BF01538132. 2831015

[cph470022-bib-0154] Sekioka, T. , M. Kadode , N. Osakada , et al. 2017. “A New CysLT(1) and CysLT(2) Receptors‐Mediated Anaphylaxis Guinea Pig Model.” Prostaglandins, Leukotrienes, and Essential Fatty Acids 119: 18–24. 10.1016/j.plefa.2017.03.002. 28410666

[cph470022-bib-0155] Seymour, M. L. , S. Rak , D. Aberg , et al. 2001. “Leukotriene and Prostanoid Pathway Enzymes in Bronchial Biopsies of Seasonal Allergic Asthmatics.” American Journal of Respiratory and Critical Care Medicine 164, no. 11: 2051–2056. 10.1164/ajrccm.164.11.2008137. 11739134

[cph470022-bib-0156] Sharma, R. K. , A. C. Oliveira , T. Yang , et al. 2020. “Gut Pathology and Its Rescue by ACE2 (Angiotensin‐Converting Enzyme 2) in Hypoxia‐Induced Pulmonary Hypertension.” Hypertension 76, no. 1: 206–216. 10.1161/HYPERTENSIONAHA.120.14931. 32418496 PMC7505091

[cph470022-bib-0157] Sharon, P. , and W. F. Stenson . 1984. “Enhanced Synthesis of Leukotriene B4 by Colonic Mucosa in Inflammatory Bowel Disease.” Gastroenterology 86, no. 3: 453–460. 6319219

[cph470022-bib-0158] Shih, D. Q. , L. Zheng , X. Zhang , et al. 2014. “Inhibition of a Novel Fibrogenic Factor Tl1a Reverses Established Colonic Fibrosis.” Mucosal Immunology 7, no. 6: 1492–1503. 10.1038/mi.2014.37. 24850426 PMC4205266

[cph470022-bib-0159] Singh, R. K. , R. Tandon , S. G. Dastidar , and A. Ray . 2013. “A Review on Leukotrienes and Their Receptors With Reference to Asthma.” Journal of Asthma: Official Journal of the Association for the Care of Asthma 50, no. 9: 922–931. 10.3109/02770903.2013.823447. 23859232

[cph470022-bib-0160] Sjoberg, L. C. , A. Z. Nilsson , Y. Lei , J. A. Gregory , M. Adner , and G. P. Nilsson . 2017. “Interleukin 33 Exacerbates Antigen Driven Airway Hyperresponsiveness, Inflammation and Remodeling in a Mouse Model of Asthma.” Scientific Reports 7, no. 1: 4219. 10.1038/s41598-017-03674-0. 28652606 PMC5484710

[cph470022-bib-0161] Sklyarov, A. Y. , N. B. Panasyuk , and I. S. Fomenko . 2011. “Role of Nitric Oxide‐Synthase and Cyclooxygenase/Lipooxygenase Systems in Development of Experimental Ulcerative Colitis.” Journal of Physiology and Pharmacology 62, no. 1: 65–73. 21451211

[cph470022-bib-0162] Song, C. , L. Zhou , Y. Xiong , et al. 2025. “Five‐Month Real‐Ambient PM(2.5) Exposure Impairs Learning in Brown Norway Rats: Insights From Multi Omics‐Based Analysis.” Ecotoxicology and Environmental Safety 294: 118065. 10.1016/j.ecoenv.2025.118065. 40147172

[cph470022-bib-0163] Songur, N. , Y. Songur , M. Tuzun , et al. 2003. “Pulmonary Function Tests and High‐Resolution CT in the Detection of Pulmonary Involvement in Inflammatory Bowel Disease.” Journal of Clinical Gastroenterology 37, no. 4: 292–298. 10.1097/00004836-200310000-00006. 14506385

[cph470022-bib-0164] Stanke‐Labesque, F. , J. Pofelski , A. Moreau‐Gaudry , G. Bessard , and B. Bonaz . 2008. “Urinary Leukotriene E4 Excretion: A Biomarker of Inflammatory Bowel Disease Activity.” Inflammatory Bowel Diseases 14, no. 6: 769–774. 10.1002/ibd.20403. 18286646

[cph470022-bib-0165] Sun, M. , R. Wang , and Q. Han . 2017. “Inhibition of Leukotriene B4 Receptor 1 Attenuates Lipopolysaccharide‐Induced Cardiac Dysfunction: Role of AMPK‐Regulated Mitochondrial Function.” Scientific Reports 7: 44352. 10.1038/srep44352. 28290498 PMC5349523

[cph470022-bib-0166] Sutherland, E. R. , E. Goleva , L. P. Jackson , A. D. Stevens , and D. Y. Leung . 2010. “Vitamin D Levels, Lung Function, and Steroid Response in Adult Asthma.” American Journal of Respiratory and Critical Care Medicine 181, no. 7: 699–704. 10.1164/rccm.200911-1710OC. 20075384 PMC2868500

[cph470022-bib-0167] Swed, S. , B. Sawaf , F. Al‐Obeidat , et al. 2024. “Asthma Prevalence Among United States Population Insights From NHANES Data Analysis.” Scientific Reports 14, no. 1: 8059. 10.1038/s41598-024-58429-5. 38580691 PMC10997649

[cph470022-bib-0168] Tahaghoghi‐Hajghorbani, S. , A. Ajami , S. Ghorbanalipoor , et al. 2019. “Protective Effect of TSLP and IL‐33 Cytokines in Ulcerative Colitis.” Auto Immun Highlights 10, no. 1: 1. 10.1186/s13317-019-0110-z. 30868311 PMC6416230

[cph470022-bib-0169] Takahashi, Y. , T. Kobayashi , C. N. D'Alessandro‐Gabazza , et al. 2019. “Protective Role of Matrix Metalloproteinase‐2 in Allergic Bronchial Asthma.” Frontiers in Immunology 10: 1795. 10.3389/fimmu.2019.01795. 31428095 PMC6687911

[cph470022-bib-0170] Takedatsu, H. , K. S. Michelsen , B. Wei , et al. 2008. “TL1A (TNFSF15) Regulates the Development of Chronic Colitis by Modulating Both T‐Helper 1 and T‐Helper 17 Activation.” Gastroenterology 135, no. 2: 552–567. 10.1053/j.gastro.2008.04.037. 18598698 PMC2605110

[cph470022-bib-0171] Tanaka, J. , N. Watanabe , M. Kido , et al. 2009. “Human TSLP and TLR3 Ligands Promote Differentiation of Th17 Cells With a Central Memory Phenotype Under Th2‐Polarizing Conditions.” Clinical and Experimental Allergy 39, no. 1: 89–100. 10.1111/j.1365-2222.2008.03151.x. 19055649 PMC7164823

[cph470022-bib-0172] Thawanaphong, S. , A. Nair , E. Volfson , P. Nair , and M. Mukherjee . 2024. “IL‐18 Biology in Severe Asthma.” Frontiers in Medicine 11: 1486780. 10.3389/fmed.2024.1486780. 39554494 PMC11566457

[cph470022-bib-0173] Thompson, M. D. , V. Capra , M. T. Clunes , et al. 2016. “Cysteinyl Leukotrienes Pathway Genes, Atopic Asthma and Drug Response: From Population Isolates to Large Genome‐Wide Association Studies.” Frontiers in Pharmacology 7: 299. 10.3389/fphar.2016.00299. 27990118 PMC5131607

[cph470022-bib-0174] Tillie‐Leblond, I. , J. Pugin , C. H. Marquette , et al. 1999. “Balance Between Proinflammatory Cytokines and Their Inhibitors in Bronchial Lavage From Patients With Status Asthmaticus.” American Journal of Respiratory and Critical Care Medicine 159, no. 2: 487–494. 10.1164/ajrccm.159.2.9805115. 9927362

[cph470022-bib-0175] Trischler, J. , C. M. Muller , S. Konitzer , et al. 2015. “Elevated Exhaled Leukotriene B(4) in the Small Airway Compartment in Children With Asthma.” Annals of Allergy, Asthma & Immunology 114, no. 2: 111–116. 10.1016/j.anai.2014.11.022. 25624130

[cph470022-bib-0176] Trivedi, R. , and K. Barve . 2020. “Gut Microbiome a Promising Target for Management of Respiratory Diseases.” Biochemical Journal 477, no. 14: 2679–2696. 10.1042/BCJ20200426. 32726437

[cph470022-bib-0177] Trompette, A. , E. S. Gollwitzer , K. Yadava , et al. 2014. “Gut Microbiota Metabolism of Dietary Fiber Influences Allergic Airway Disease and Hematopoiesis.” Nature Medicine 20, no. 2: 159–166. 10.1038/nm.3444. 24390308

[cph470022-bib-0178] Turner‐Warwick, M. 1968. “Fibrosing Alveolitis and Chronic Liver Disease.” Quarterly Journal of Medicine 37, no. 145: 133–149. 4872529

[cph470022-bib-0179] Tzanakis, N. , D. Bouros , M. Samiou , et al. 1998. “Lung Function in Patients With Inflammatory Bowel Disease.” Respiratory Medicine 92, no. 3: 516–522. 10.1016/s0954-6111(98)90301-8. 9692115

[cph470022-bib-0180] Tzanakis, N. E. , I. G. Tsiligianni , and N. M. Siafakas . 2010. “Pulmonary Involvement and Allergic Disorders in Inflammatory Bowel Disease.” World Journal of Gastroenterology 16, no. 3: 299–305. 10.3748/wjg.v16.i3.299. 20082474 PMC2807949

[cph470022-bib-0181] Underhill, D. M. , and I. D. Iliev . 2014. “The Mycobiota: Interactions Between Commensal Fungi and the Host Immune System.” Nature Reviews. Immunology 14, no. 6: 405–416. 10.1038/nri3684. PMC4332855 24854590

[cph470022-bib-0182] Valacer, D. J. 1999. “New Treatments for Asthma: The Role of Leukotriene Modifier Agents.” Journal of the National Medical Association 91, no. 8 Suppl: 26S–39S. 12653390 PMC2608482

[cph470022-bib-0183] Virta, L. J. , M. Ashorn , and K. L. Kolho . 2013. “Cow's Milk Allergy, Asthma, and Pediatric IBD.” Journal of Pediatric Gastroenterology and Nutrition 56, no. 6: 649–651. 10.1097/MPG.0b013e318285e9d8. 23319082

[cph470022-bib-0184] Waddell, A. , J. E. Vallance , S. Fox , and M. J. Rosen . 2021. “IL‐33 Is Produced by Colon Fibroblasts and Differentially Regulated in Acute and Chronic Murine Colitis.” Scientific Reports 11, no. 1: 9575. 10.1038/s41598-021-89119-1. 33953267 PMC8100152

[cph470022-bib-0185] Wallace, J. L. , W. K. MacNaughton , G. P. Morris , and P. L. Beck . 1989. “Inhibition of Leukotriene Synthesis Markedly Accelerates Healing in a Rat Model of Inflammatory Bowel Disease.” Gastroenterology 96, no. 1: 29–36. 10.1016/0016-5085(89)90760-9. 2535830

[cph470022-bib-0186] Wang, Z. , L. F. Cuthbertson , C. Thomas , et al. 2024. “IL‐1alpha Is Required for T Cell‐Driven Weight Loss After Respiratory Viral Infection.” Mucosal Immunology 17, no. 2: 272–287. 10.1016/j.mucimm.2024.02.005. 38382577 PMC11009121

[cph470022-bib-0187] Weiss, J. W. , J. M. Drazen , N. Coles , et al. 1982. “Bronchoconstrictor Effects of Leukotriene C in Humans.” Science 216, no. 4542: 196–198. 10.1126/science.7063880. 7063880

[cph470022-bib-0188] Welsh, K. G. , K. Rousseau , G. Fisher , et al. 2017. “MUC5AC and a Glycosylated Variant of MUC5B Alter Mucin Composition in Children With Acute Asthma.” Chest 152, no. 4: 771–779. 10.1016/j.chest.2017.07.001. 28716644 PMC5624091

[cph470022-bib-0189] Wu, X. , S. Wei , M. Chen , et al. 2022. “P2RY13 Exacerbates Intestinal Inflammation by Damaging the Intestinal Mucosal Barrier via Activating IL‐6/STAT3 Pathway.” International Journal of Biological Sciences 18, no. 13: 5056–5069. 10.7150/ijbs.74304. 35982893 PMC9379400

[cph470022-bib-0190] Wu, Y. , C. Cui , F. F. Bi , et al. 2022. “Montelukast, Cysteinyl Leukotriene Receptor 1 Antagonist, Inhibits Cardiac Fibrosis by Activating APJ.” European Journal of Pharmacology 923: 174892. 10.1016/j.ejphar.2022.174892. 35358494

[cph470022-bib-0191] Xiong, T. , X. Zheng , K. Zhang , et al. 2022. “Ganluyin Ameliorates DSS‐Induced Ulcerative Colitis by Inhibiting the Enteric‐Origin LPS/TLR4/NF‐kappaB Pathway.” Journal of Ethnopharmacology 289: 115001. 10.1016/j.jep.2022.115001. 35085745

[cph470022-bib-0192] Yilmaz, A. , N. Yilmaz Demirci , D. Hosgun , et al. 2010. “Pulmonary Involvement in Inflammatory Bowel Disease.” World Journal of Gastroenterology 16, no. 39: 4952–4957. 10.3748/wjg.v16.i39.4952. 20954282 PMC2957604

[cph470022-bib-0193] Yokomizo, T. , T. Izumi , K. Chang , Y. Takuwa , and T. Shimizu . 1997. “A G‐Protein‐Coupled Receptor for Leukotriene B4 That Mediates Chemotaxis.” Nature 387, no. 6633: 620–624. 10.1038/42506. 9177352

[cph470022-bib-0194] Yokomizo, T. , K. Kato , K. Terawaki , T. Izumi , and T. Shimizu . 2000. “A Second Leukotriene B(4) Receptor, BLT2. A New Therapeutic Target in Inflammation and Immunological Disorders.” Journal of Experimental Medicine 192, no. 3: 421–432. 10.1084/jem.192.3.421. 10934230 PMC2193217

[cph470022-bib-0195] Yokoyama, A. , N. Kohno , S. Fujino , et al. 1995. “Circulating Interleukin‐6 Levels in Patients With Bronchial Asthma.” American Journal of Respiratory and Critical Care Medicine 151, no. 5: 1354–1358. 10.1164/ajrccm.151.5.7735584. 7735584

[cph470022-bib-0196] Zaitsu, M. , Y. Hamasaki , M. Matsuo , T. Ichimaru , I. Fujita , and E. Ishii . 2003. “Leukotriene Synthesis Is Increased by Transcriptional Up‐Regulation of 5‐Lipoxygenase, Leukotriene A4 Hydrolase, and Leukotriene C4 Synthase in Asthmatic Children.” Journal of Asthma: Official Journal of the Association for the Care of Asthma 40, no. 2: 147–154. 10.1081/jas-120017985. 12765316

[cph470022-bib-0197] Zhang, L. , Y. Zhang , X. Jiang , et al. 2023. “Disruption of the Lung‐Gut‐Brain Axis Is Responsible for Cortex Damage Induced by Pulmonary Exposure to Zinc Oxide Nanoparticles.” Toxicology 485: 153390. 10.1016/j.tox.2022.153390. 36535435

[cph470022-bib-0198] Zhao, J. , J. Zhang , S. Tang , et al. 2021. “The Different Functions of Short and Long Thymic Stromal Lymphopoietin Isoforms in Autophagy‐Mediated Asthmatic Airway Inflammation and Remodeling.” Immunobiology 226, no. 5: 152124. 10.1016/j.imbio.2021.152124. 34333403

[cph470022-bib-0199] Zheng, Y. , L. Bonfili , T. Wei , and A. M. Eleuteri . 2023. “Understanding the Gut‐Brain Axis and Its Therapeutic Implications for Neurodegenerative Disorders.” Nutrients 15, no. 21: 4631. 10.3390/nu15214631. 37960284 PMC10648099

[cph470022-bib-0200] Zhou, L. , J. Zhang , X. Han , et al. 2022. “CysLT(2)R Antagonist HAMI 3379 Ameliorates Post‐Stroke Depression Through NLRP3 Inflammasome/Pyroptosis Pathway in Gerbils.” Brain Sciences 12, no. 8: 976. 10.3390/brainsci12080976. 35892417 PMC9330558

[cph470022-bib-0201] Zhuang, W. , and Z. Li . 2023. “Antibody Targeting TSLP Suppresses DSS‐Induced Colitis and Activation of the JAK2/STAT5 Pathway in Mice.” European Cytokine Network 34, no. 4: 46–53. 10.1684/ecn.2023.0489. 38526174

[cph470022-bib-0202] Ziegler, S. F. , and D. Artis . 2010. “Sensing the Outside World: TSLP Regulates Barrier Immunity.” Nature Immunology 11, no. 4: 289–293. 10.1038/ni.1852. 20300138 PMC2924817

[cph470022-bib-0203] Zou, X. , R. L. Lu , B. Liao , S. J. Liu , and S. X. Dai . 2023. “Causal Relationship Between Asthma and Ulcerative Colitis and the Mediating Role of Interleukin‐18: A Bidirectional Mendelian Study and Mediation Analysis.” Frontiers in Immunology 14: 1293511. 10.3389/fimmu.2023.1293511. 38162651 PMC10757619

[cph470022-bib-0204] Zovko, A. , E. Yektaei‐Karin , D. Salamon , A. Nilsson , J. Wallvik , and L. Stenke . 2018. “Montelukast, a Cysteinyl Leukotriene Receptor Antagonist, Inhibits the Growth of Chronic Myeloid Leukemia Cells Through Apoptosis.” Oncology Reports 40, no. 2: 902–908. 10.3892/or.2018.6465. 29845257

